# An Intronic *Flk1* Enhancer Directs Arterial-Specific Expression via RBPJ-Mediated Venous Repression

**DOI:** 10.1161/ATVBAHA.116.307517

**Published:** 2016-05-25

**Authors:** Philipp W. Becker, Natalia Sacilotto, Svanhild Nornes, Alice Neal, Max O. Thomas, Ke Liu, Chris Preece, Indrika Ratnayaka, Benjamin Davies, George Bou-Gharios, Sarah De Val

**Affiliations:** From the Ludwig Institute for Cancer Research, Nuffield Department of Clinical Medicine (P.W.B., N.S., S.N., A.N., M.O.T., I.R., S.D.V.) and The Wellcome Trust Centre for Human Genetics (C.P., B.D.), University of Oxford, Oxford, United Kingdom; and Institute of Ageing and Chronic Disease, Faculty of Health and Life Sciences, University of Liverpool, Liverpool, United Kingdom (K.L., G.B.-G.).

**Keywords:** arterial-venous specification, artery, endothelial cells, mice, notch, veins, zebrafish

## Abstract

**Objective—:**

The vascular endothelial growth factor (VEGF) receptor Flk1 is essential for vascular development, but the signaling and transcriptional pathways by which its expression is regulated in endothelial cells remain unclear. Although previous studies have identified 2 *Flk1* regulatory enhancers, these are dispensable for Flk1 expression, indicating that additional enhancers contribute to *Flk1* regulation in endothelial cells. In the present study, we sought to identify *Flk1* enhancers contributing to expression in endothelial cells.

**Approach and Results—:**

A region of the 10th intron of the *Flk1* gene (Flk1in10) was identified as a putative enhancer and tested in mouse and zebrafish transgenic models. This region robustly directed reporter gene expression in arterial endothelial cells. Using a combination of targeted mutagenesis of transcription factor–binding sites and gene silencing of transcription factors, we found that Gata and Ets factors are required for Flk1in10 enhancer activity in all endothelial cells. Furthermore, we showed that activity of the Flk1in10 enhancer is restricted to arteries through repression of gene expression in venous endothelial cells by the Notch pathway transcriptional regulator Rbpj.

**Conclusions—:**

This study demonstrates a novel mechanism of arterial–venous identity acquisition, indicates a direct link between the Notch and VEGF signaling pathways, and illustrates how *cis*-regulatory diversity permits differential expression outcomes from a limited repertoire of transcriptional regulators.

## Abstract 2

Supplemental Digital Content is available in the text.

The formation of the vascular system begins with vasculogenesis, the coalescence of endothelial precursor cells to form the primitive dorsal aortae, cardinal veins, and the capillary plexus of the yolk sac and embryo.^1^ This primitive vascular network then expands, remodels, and matures into a hierarchically organized network of arteries, capillaries, and veins.^2^ Although the phenotypic and genetic differences between endothelial cells in different branches of the vasculature were initially thought to be downstream of hemodynamic forces, it is now clear that arteriovenous differentiation of endothelial cells occurs before the onset of blood flow. Notch signaling can activate early arterial gene expression via the Notch transcriptional effector Rbpj (CSL) and SoxF factors, suggesting that arterial identity has to be acquired and that the default state of endothelial cells is venous.^3,4^ However, the vein-specific orphan nuclear receptor COUP-TFII (Nr2f2) has been shown to repress arterial gene expression,^5^ and the promotion of venous identity downstream of PI3K (phosphoinositide 3-kinase) occurs through inhibition of proarterial pathways.^6^ Consequently, the degree to which particular endothelial cell identities are actively acquired or repressed may vary and remains poorly understood.

The vascular endothelial growth factor (VEGF) pathway is crucial for vascular development,^7^ mediating various processes in endothelial cells, including sprouting, migration, proliferation, and differentiation. VEGF signaling has also been implicated in arterial differentiation upstream of Notch, although VEGF receptors are found in both arterial and venous endothelial cells.^8^ In mammals, Flk1 (also known as VEGFR2/KDR), a tyrosine kinase receptor, is the main mediator of angiogenic growth in response to VEGF-A.^7^ Flk1^−/−^ mice die before midgestation with severe vascular and hematopoietic defects and lack mature endothelial cells.^9^ Zebrafish and other teleosts contain 2 orthologues to Flk1, *kdr* (kdrb) and *kdrl* (kdra).^10^ Although it is *kdr* that shares direct lineage with mouse Flk1, *kdrl* seems more crucial for zebrafish vascular patterning, suggesting that although the fundamentals of VEGF signaling are conserved between the 2 model systems, the specific roles for each receptor may have diverged.^11^

Previous studies identified a pan-endothelial enhancer within the first intron of *Flk1* (Flk1intron1)^12^; however, deletion of this enhancer had no detectable effect on *Flk1* expression.^13^ A second Flk1 enhancer (Flk1 dorsal multipotent mesodermal enhancer), located 5′ upstream of *Flk1*,^14^ directs expression in the lateral mesoderm during early embryogenesis but not in differentiated endothelial cells.^14^ Consequently, it is likely that additional enhancer elements contribute to Flk1 activity in endothelial cells, similar to the multiple endothelial-specific enhancers regulating the *Mef2c*, *Tal1*, and *Dll4* loci.^4,15^ These arrangements of enhancers, binding a varied array of transcription factors downstream of multiple signaling inputs, may explain the considerable phenotypic heterogeneity of endothelial cells among tissues, developmental stages, and angiogenic status.

Many different signaling cascades, including Wnt, transforming growth factor-β, and Notch, interact with the VEGF pathway at multiple stages of vascular development, yet these interactions are multifaceted, context dependent, and affect gene expression patterns both directly and indirectly.^16–18^ Given the crucial role of VEGF signaling in both developmental and tumor angiogenesis, a more complete understanding of the manner in which components of the VEGF pathway are regulated is key to our understanding of how vascular signaling pathways interact to pattern the embryonic vasculature correctly, as well as our ability to modulate vessel growth accurately in pathological conditions.

## Materials and Methods

Materials and Methods are available in the online-only Data Supplement.

## Results

### Identification of an Arterial-Restricted Endothelial Cell Enhancer Within the 10th Intron of the Flk1 Gene

**Figure 1.**
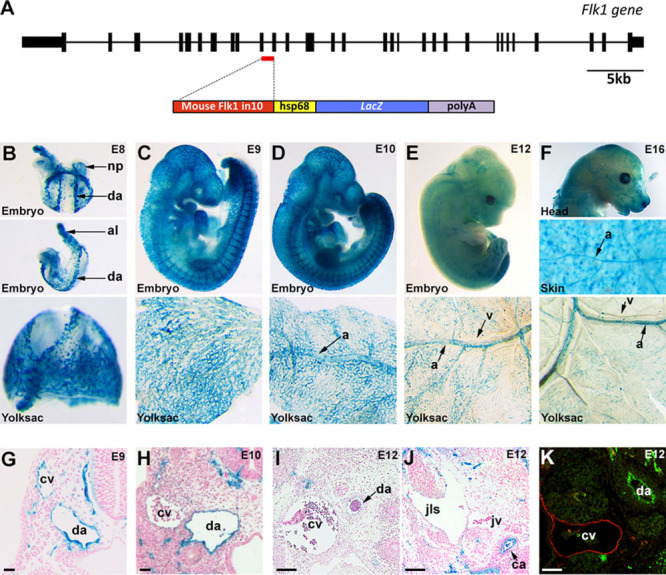
The mouse Flk1in10 enhancer directs arterial-restricted expression in transgenic mice. **A**, Schematic representation of the mouse Flk1in10 enhancer (top line, exons are black boxes) and Flk1in10:*LacZ* transgene (bottom line). **B–K**, The Flk1in10:*LacZ* transgene directs arterial expression. Representative whole-mount embryos and yolk sac tissue from the Flk1in10:*LacZ* transgenic line (**B–F**) show reporter gene expression (X-gal staining, blue) in the vasculature from embryonic day 8 (E8) to E16. X-gal staining is initially detected throughout the vasculature but becomes restricted to the arterial compartment during development. **G–J**, In transverse sections through E9–E12 transgenic embryos, X-gal staining is detected in both cardinal vein and dorsal aorta at E9 (**G**) but is stronger in the dorsal aorta by E10 and is not detected in the venous or lymphatic vasculature by E12. **K**, E12 transverse paraffin sections showed that expression of the venous marker endomucin does not overlap with that of the β-galactosidase reporter gene. a indicates artery; al, allantois; ca, carotid artery; cv, cardinal vein; da, dorsal aorta; jls, jugular lymph sac; jv, jugular vein; np, neural plexus; and v, vein.

An in silico search of the mouse and human Flk1/KDR loci for conserved sequences enriched in enhancer-associated histone modifications and endothelial cell–specific DNaseI hypersensitivity sites identified the 10th intron of Flk1 (Flk1in10) as a putative enhancer region (Figure I in the online-only Data Supplement^19^). To verify and characterize the activity of this region, the 825-bp mouse Flk1in10 sequence was cloned upstream of the silent hsp68 minimal promoter and *LacZ* reporter gene and used to generate the stable transgenic mouse line Flk1in10:*LacZ* (Figure 1A). Analysis of embryonic Flk1in10:*LacZ* mice clearly demonstrated enhancer activity in the developing vasculature and heart (Figure 1B–1K; Figure IIA in the online-only Data Supplement). At embryonic day (E) 9, activity of the Flk1in10 enhancer was detected throughout the endothelial cells of the vasculature, including both venous and arterial compartments (Figure 1C and 1G). However, in E10 embryos, enhancer activity was stronger in arterial endothelial cells, and by E12, *LacZ* reporter gene expression was only detected in the arterial vasculature. Arterial vascular expression was maintained throughout development, although expression was absent in certain tissue beds, most notably the lung (Figure 1D–1F and 1H–1K; Figure IIB in the online-only Data Supplement). Furthermore, transgene expression decreased after birth and was absent in adult organs (Figure IIB in the online-only Data Supplement). These results indicate that the Flk1in10 sequence represents a developmental endothelial enhancer which becomes restricted to the arterial compartment.

**Figure 2.**
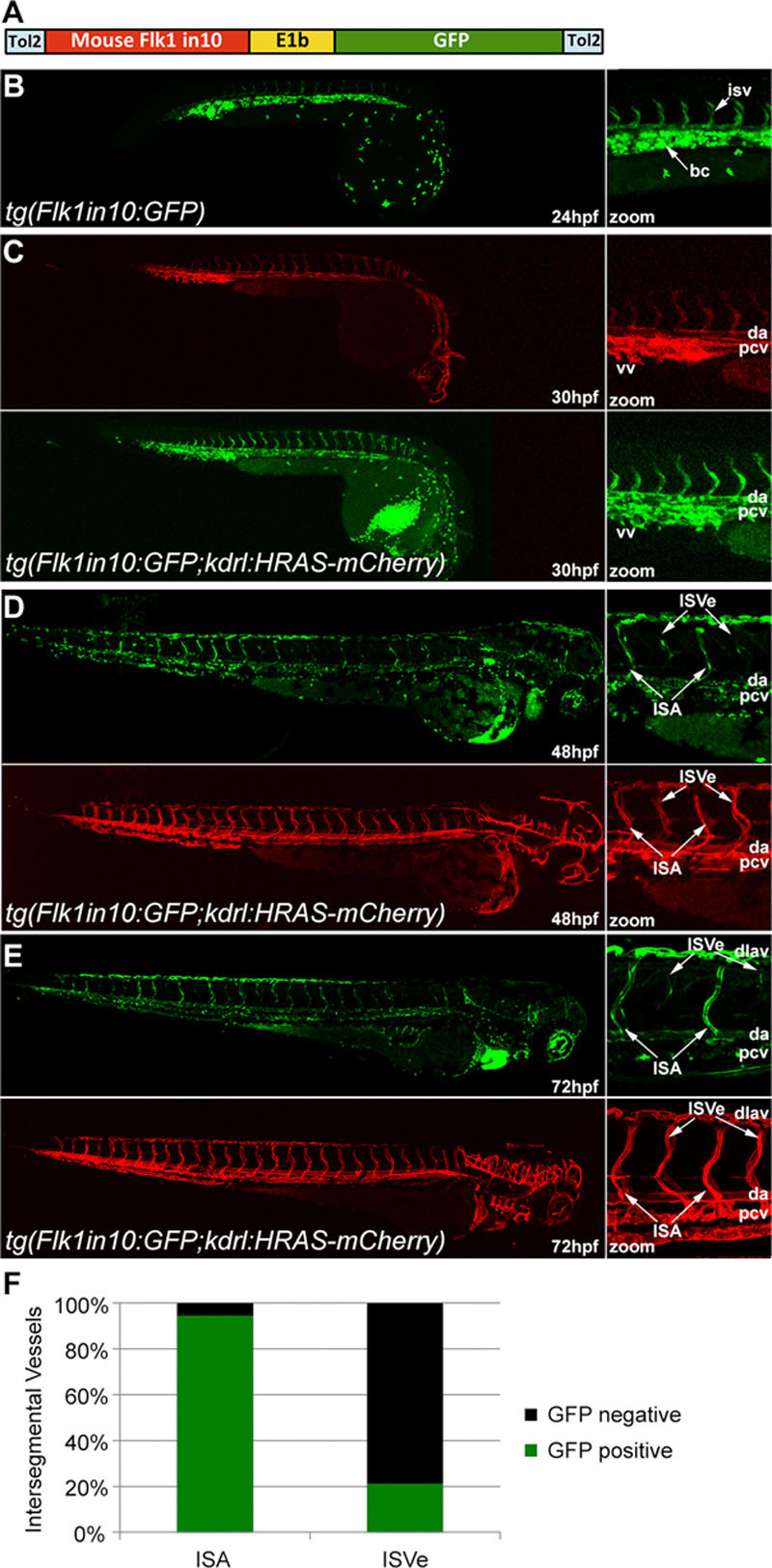
The mouse Flk1in10 enhancer directs arterial-restricted expression in transgenic zebrafish. **A**, Schematic representation of the Flk1in10:GFP transgene. **B**, The mouse Flk1in10:GFP transgene directs endothelial cell–specific expression in transgenic zebrafish line *tg*(*Flk1in10:GFP*) at 24 h post fertilization (hpf). **C–E**, *Tg*(*Flk1in10:GFP;kdrl:HRAS:mCherry*) zebrafish expresses GFP in most endothelial cells at 30 hpf (**C**). At 48 hpf, GFP expression in the axial vessels becomes restricted to the dorsal aorta and to a subset of intersegmental vessels, corresponding to the developing intersegmental arteries (ISA; **D**; see also Movie I in the online-only Data Supplement). At 72 hpf, little dorsal aorta expression could be detected, while GFP expression is maintained in the ISA and dorsal longitudinal anastomotic vessel (**E**). **F**, Bar chart detailing GFP expression pattern in the ISA and intersegmental veins (ISVe) at 72 hpf. Represents a total of 18 embryos, ISA and ISVe identity established by using kdrl:HRAS-mCherry expression to determine whether each vessel connected to dorsal aorta or posterior cardinal vein. Any detectable level of GFP expression constituted positive, and Figure III in the online-only Data Supplement details analysis methods. bc indicates blood cells; da, dorsal aorta; dlav, dorsal longitudinal anastomotic vessel; dv, dorsal vein; GFP, green fluorescent protein; isv, intersegmental vessels; ISVe, intersegmental vein; and vv, ventral vein.

To test whether the mouse Flk1in10 enhancer sequence also drives endothelial expression in transgenic zebrafish, it was cloned upstream of the E1b minimal promoter^20^ and green fluorescent protein (GFP) reporter gene (Figure 2A) and used to generate the *tg*(*Flk1in10:GFP*) transgenic fish line. This line was then crossed with the pan-endothelial *tg(kdrl:HRAS-mCherry*) line^21^ to enable analysis of GFP expression patterns within the vasculature (Figure 2B–2F). GFP expression was detected in the axial, intersegmental, and head vessels; blood cells; and the heart, suggesting that the transcriptional pathways regulating mouse Flk1in10 are conserved in zebrafish. At 48 hours post fertilization, reporter gene expression within the axial vessels diminished and was detected weakly only in the dorsal aorta. Furthermore, GFP expression became restricted to efferent intersegmental vessels (Figure 2D; Movie I in the online-only Data Supplement). Restriction of the transgene to the intersegmental arteries but not intersegmental veins was also clearly detected at 72 hours post fertilization, although expression was now mostly absent in the axial vessels (Figure 2E and 2F; Figure III in the online-only Data Supplement). To rule out a role for flow in the establishment of this expression pattern, transgene expression was investigated in embryos lacking heart function. Although the absence of flow resulted in a decrease in GFP intensity, arterial restriction in the intersegmental vessels was still observed (Figure IV in the online-only Data Supplement).

These results demonstrate that Flk1in10 represents a novel, arterial-restricted gene enhancer, unlike the Flk1 intron1 enhancer, which directs pan-vascular expression^12^ (Figure V in the online-only Data Supplement). This supports the hypothesis that regulation of Flk1 in the vasculature is achieved by several modular enhancer elements and suggests that VEGF receptor availability can be regulated independently in different endothelial subpopulations. Although endogenous mouse *Flk1* mRNA is found in both arterial and venous endothelial cells during embryonic development,^9^ interactome studies mapping gene regulatory domains have clearly demonstrated the association of widely expressed genes with multiple cell type–specific enhancers,^22^ and endothelial subtype–specific enhancers have been reported for other pan-vascular transcribed genes.^23^

### Flk1in10 Enhancer Contains Functional Binding Motifs for Ets, Gata, Rbpj, SoxF, and FoxC Transcription Factors

**Figure 3.**
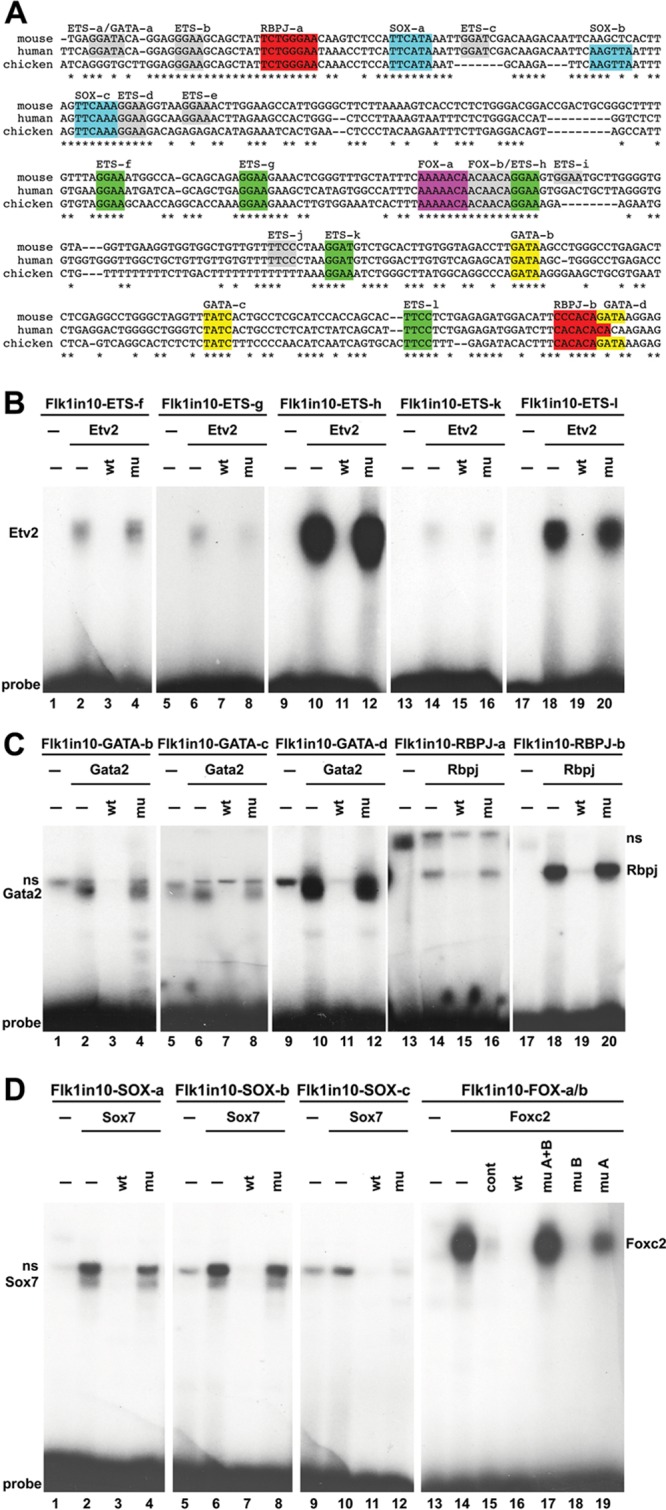
The Flk1in10 enhancer contains *cis*-motifs that bind Etv2, Gata2, Rbpj, Sox7, and Foxc2 transcription factors. **A**, Multispecies alignment of the conserved region of the Flk1in10 enhancer. Colored sequences depict confirmed consensus binding motifs, and gray sequences depict motifs that did not bind in electrophoretic mobility shift assay. **B**, Radiolabeled oligonucleotide probes encompassing Flk1in10 ETS-f (lanes 1–4), ETS-g (lanes 5–8), ETS-h (lanes 9–12), ETS-k (lanes 13–16), and ETS-l motifs (lanes 17–20) were bound to recombinant Etv2 proteins. All proteins efficiently bound to labeled probes (lanes 2, 6, 10, 14, and 18) were competed by excess unlabeled self-probe (wt, lanes 3, 7, 11, 15, and 19) but not by mutant self-probe (mu, lanes 4, 8, 12, 16, and 20). **C**, Radiolabeled oligonucleotide probes encompassing Flk1in10 GATA-b (lanes 1–4), GATA-c (lanes 5–8), GATA-d (lanes 9–12), RBPJ-a (lanes 13–16), and RBPJ-b motifs (lanes 17–20) were bound to recombinant Gata2 and Rbpj proteins. All proteins efficiently bound to labeled probes (lanes 2, 6, 10, 14, and 18) were competed by excess unlabeled self-probe (wt, lanes 3, 7, 11, 15, and 19) but not by mutant self-probe (mu, lanes 4, 8, 12, 16, and 20). **D**, Radiolabeled oligonucleotide probes encompassing Flk1in10 SOX-a (lanes 1–4), SOX-b (lanes 5–8), SOX-c (lanes 9–12), and FOX-a/b (lanes 13–19) were bound to recombinant Sox7 and Foxc2 proteins. All proteins efficiently bound to labeled probes (lanes 2, 6, 10, and 14) were competed by excess unlabeled self-probe (wt, lanes 3, 7, 11, and 16) but not by mutant self-probe (mu, lanes 4, 8, and 12). For FOX-a/b, mutant self-probe with mutations to both FOX motifs (mu A+B) or to FOX-a could not compete with labeled probes (17, 19), whereas a mutant self-probe with mutations to FOX-b (mu B) was still able to robustly compete with labeled probe (18). mu indicates mutant; ns, nonspecific binding; and wt, wild-type.

The mouse Flk1in10 enhancer shares significant sequence conservation between mammals and birds, but no detectable conservation to reptiles or fish (Figure IA and IB in the online-only Data Supplement). We used ClustalW analysis^24^ to analyze the conserved enhancer sequences and identified conserved putative binding motifs for Ets, Gata, Rbpj, Sox, and Fox, which were then functionally verified by electrophoretic mobility shift assays (EMSAs) using members of each transcription factor family known to be expressed in endothelial cells (Figure 3A–3D). Additional, less conserved motifs for Ets (ETS-a, c, e, i, and j), Gata (GATA-a and d), and Rbpj (RBPJ-b) were also tested in EMSA to ensure that functional yet poorly conserved binding sites were not overlooked. Five putative ETS motifs directly bound the Ets factor Etv2 (ETS-f, g, h, k, and l; Figure 3B), and 3 of 4 GATA motifs within the conserved enhancer directly bound Gata2 (GATA-b, c, and d; Figure 3C). All 3 putative SOX motifs directly bound Sox7 (SOX-a, b, and c), and both putative RBPJ motifs directly bound the protein (Figure 3C and 3D). The ETS-h motif was directly adjacent to a putative Forkhead binding site (FOX-b), forming a FOX:ETS motif (Figure 3).^25^ However, although a composite oligo containing both FOX-a and b motifs bound Foxc2, mutations to the FOX-b site did not ablate this binding (Figure 3D), suggesting that only the distal FOX-a site was required for Foxc2 binding and that the putative FOX:ETS motif may not be functional.

### Flk1in10 Enhancer Activity Requires Both ETS and GATA Transcription Factors

**Figure 4.**
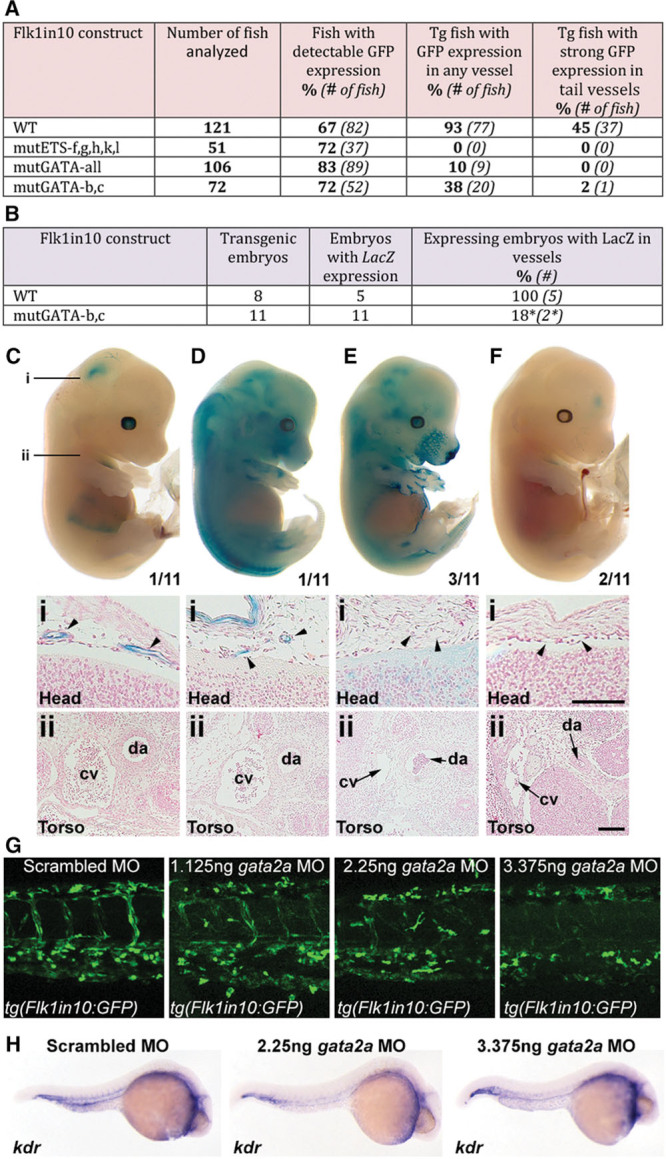
The Flk1in10 enhancer requires ETS and GATA motifs for endothelial expression. **A** and **B**, Summary of reporter gene expression detected in (**A**) 48 hpf Tol2-mediated mosaic transient transgenic zebrafish embryos and (**B**) E12 transient transgenic mice. Asterisk indicates weak staining in head region only. **C–F**, Whole-mount (**top**) and transverse sections (**bottom**) of representative E12 X-gal–stained embryos transgenic for the Flk1in10mutGATA-b,c construct. Depicted sections correspond to the embryos shown. Number in bottom corner of whole-mount embryo denotes the number of embryos similar to that shown. Lines marked i and ii on **C** mark the approximate location of transverse sections. Arrowheads mark head vessels. **G**, Analysis of scrambled, 1.125 ng, 2.25 ng, and 3.375 ng *gata2a* morpholino (MO) in 48 h post fertilization (hpf) *tg*(*Flk1in10:GFP*) embryos. **H**, Analysis of scrambled, 2.25 ng and 3.375 ng *gata2a* MO in 26 hpf WT zebrafish embryos, using whole-mount *kdr* in situ hybridization. cv indicates cardinal vein; da, doral aorta; and GFP, green fluorescent protein.

Several Ets transcription factors play crucial roles in vascular specification, and all described endothelial cell enhancers contain multiple ETS-binding motifs.^15^ To confirm that Ets binding was required for Flk1in10 activity, all 5 functional ETS motifs were mutated (sequence information is available in the Materials and Methods in the online-only Data Supplement; EMSA was used to validate all mutations), and activity of the modified mouse enhancer (mutETS-f, g, h, k, and l) was tested in transgenic zebrafish (Figure 4A). As expected, the ablation of the functional ETS motifs resulted in a total loss of enhancer activity in the vasculature, supporting the essential role of Ets factors in vascular gene activation.

Both the Flk1 intron1 and dorsal multipotent mesodermal enhancers also contain conserved GATA motifs,^12,14^ and enforced Gata2 expression can induce Flk1 expression in hemangioblast culture conditions.^26^ To study the potential role for Gata transcription factors in the direct activation of the Flk1in10 enhancer, we mutated all GATA-binding motifs within the enhancer and tested the activity of the modified enhancer (mutGATA-all) in transgenic zebrafish, analyzing multiple transient transgenic embryos to overcome any variability from random transgene integration (Figure 4A). Mutation of all GATA motifs resulted in a severe reduction in vascular enhancer activity, and no transgenic fish expressed the reporter gene robustly in the vasculature (Figure 4A). Furthermore, mutation of just the 2 conserved motifs, GATA-b and GATA-c (mutGATA-b,c), resulted in similar patterns of expression, although a low percentage of transgenic fish had detectable enhancer activity in intersegmental vessels (Figure 4A). To confirm these observations, we generated 11 mouse embryos transgenic for the Flk1in10mutGATA-b,c construct (Figure 4B–4F). All transgenic embryos showed detectable enhancer activity, but vascular expression was nearly entirely ablated in these embryos, limited to weak expression in a small number of vessels within the head region of 2 embryos (Figure 4C and 4D). In the remaining 9 transgenic embryos, enhancer activity was completely restricted to nonvascular tissues (Figure 4E and 4F).

We next examined the consequences of morpholino knockdown of the zebrafish gene *gata2a* on the expression of the *tg(Flk1in10:GFP*) fish line (Figure 4G and 4H). *Gata2a* homozygous mutant zebrafish display defective trunk circulation and aorta morphogenesis defects, suggesting a role for Gata2 in arterial differentiation,^27^ although arterial and venous markers were unaffected.^27^ Knockdown of *gata2a* in *tg(Flk1in10:GFP*) transgenic zebrafish resulted in reduction (at low morpholino concentration) and ablation (at high morpholino concentration) of reporter gene expression (Figure 4G). Similar to *gata2a* mutant fish, arterial and venous differentiation occurred normally after morpholino-induced *gata2a* knockdown, and arterial–venous identity was maintained (Figure VI in the online-only Data Supplement). Because *kdr*, not *kdrl*, is the evolutionarily conserved orthologue of mouse Flk1, we also investigated whether *gata2a* depletion altered *kdr* expression. Similar to previous reports for *kdrl* (the functional equivalent of Flk1 in fish), expression of *kdr* was notably reduced, but not ablated, after *gata2a* depletion (Figure 4H). Conversely, knockdown of the related *gata1a* gene did not affect reporter gene expression (Figure VI in the online-only Data Supplement). These results support a requirement for Gata transcription factor binding for vascular-specific activity of the Flk1in10 enhancer.

### Arterial Restriction of Flk1in10 Enhancer Activity Is Achieved by RBPJ-Mediated Repression in Veins

**Figure 5.**
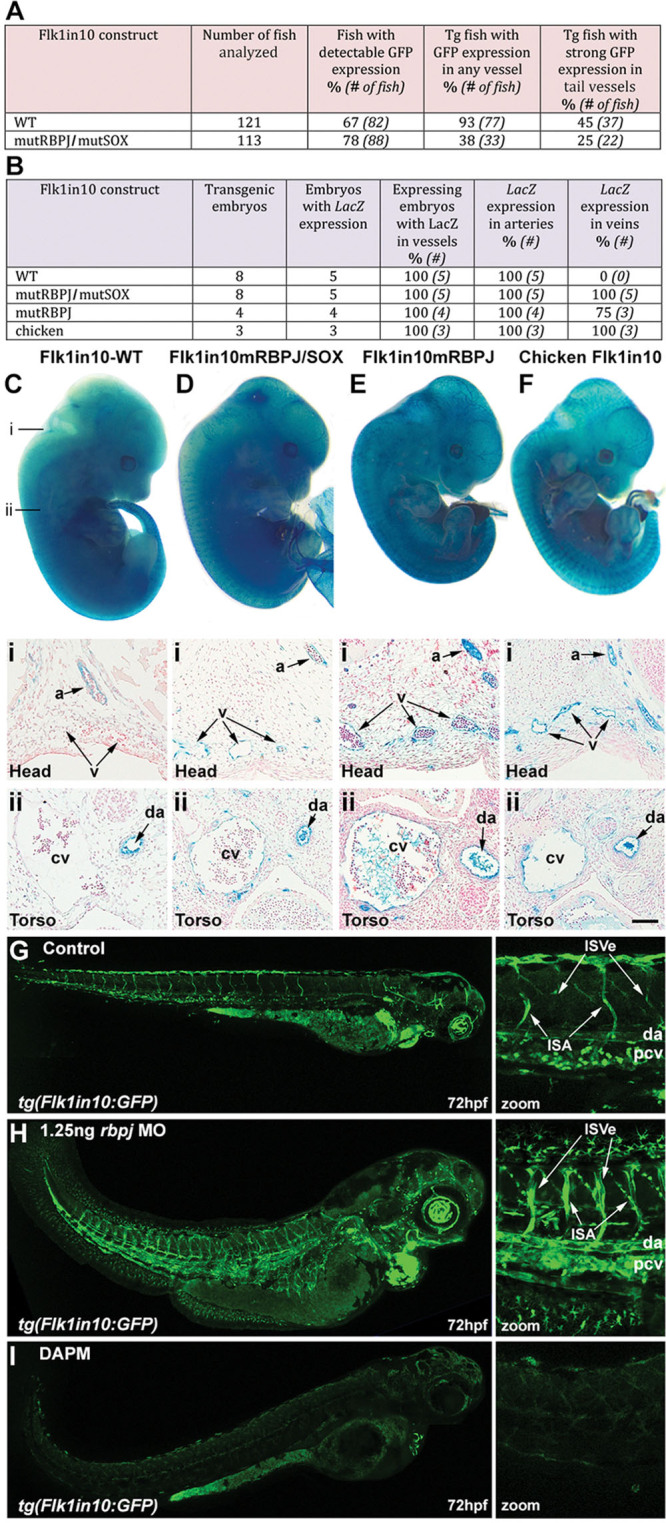
Rbpj is required for arterial restriction of the Flk1in10 enhancer. **A and B**, Summary of reporter gene expression detected in (**A**) 48 h post fertilization (hpf) Tol2-mediated mosaic transient transgenic zebrafish embryos and (**B**) embryonic day (E) 12 transient transgenic mice. **C–F**, Whole-mount (**top**) and transverse sections (**bottom**) of representative E12 X-gal–stained transient transgenic embryos expressing mouse Flk1in10 WT (**C**), mouse Flk1in10mRBPJ/SOX (**D**), mouse Flk1in10mRBPJ (**E**), and chicken Flk1in10 WT (**F**). Lines marked i and ii on **C** mark the approximate location of transverse sections in **C–F**. a indicates artery; cv, cardinal vein; da, dorsal aorta; and v, veins. **G–I**, Analysis of the effects of scrambled (**G**), 1.25 ng *rbpj* morpholino (MO) oligonucleotides (**H**), and Notch signaling inhibitor N-[N-3,5-difluorophenacetyl]-l-alanyl-S-phenylglycine methyl ester (DAPM) (**I**) in 72 h post fertilization (hpf) *tg*(*Flk1in10:GFP*) embryos. MO-mediated knockdown of *rbpj* results in expansion of Flk1in10:GFP expression into the caudal vein, the posterior cardinal vein, and intersegmental vein. da indicates dorsal aorta; GFP, green fluorescent protein; ISA, intersegmental artery; ISVe, intersegmental vein; and pcv, posterior cardinal vein.

Ets and Gata transcription factors are expressed throughout the vasculature^28,29^ and regulate many enhancers active in both arterial and venous endothelial cells.^25^ Consequently, we hypothesized that additional binding motifs within Flk1in10 may be responsible for the arterial restriction of enhancer activity. We have previously demonstrated that activity of the arterial-specific *Dll4* enhancer, which is regulated by Notch intracellular domain (NICD) and SoxF factors, requires functional binding motifs for both Rbpj and SoxF transcription factors.^4^ Therefore, we investigated the role of the RBPJ- and SOXF-binding motifs in the Flk1in10 enhancer. Surprisingly, unlike for the Dll4 enhancer, mutations to all RBPJ and SOX motifs (mutRBPJ;mutSox) did not result in the ablation of transgene expression in transgenic zebrafish and mice (Figure 5A–5D). However, in contrast to E12 mice transgenic for the Flk1in10 wild-type (Flk1in10-WT) enhancer, in which enhancer activity was restricted to arterial endothelial cells, the Flk1in10mutRBPJ/mutSox enhancer directed reporter gene expression to both arterial and venous endothelial cells. These results suggest that either the RBPJ or SOX motifs bind factors that repress the activity of the Flk1in10 enhancer in venous endothelial cells.

Because SoxF transcription factors are primarily arterial in mammals,^30^ we tested a second construct in which only the RBPJ sites were mutated (mutRBPJ). Again, expansion of enhancer activity into venous endothelial cells was seen in the majority of transgenic mice, suggesting that venous repression of Flk1in10 was mediated by Rbpj binding (Figure 5E). Of the 2 functional RBPJ motifs in the Flk1in10 enhancer, RBPJ-b bound most robustly in EMSA (Figure 3C). However, this motif was not well conserved in the Flk1in10 chicken enhancer sequence, and the orthologous chicken RBPJ-b motif could not directly bind RBPJ in EMSA (Figure VIIA in the online-only Data Supplement). Therefore, we also tested the activity of the orthologous chicken Flk1in10 enhancer. Similar to mouse Flk1in10mutRBPJ, the chicken enhancer was robustly active in both arterial and venous endothelial cells (Figure 5F; Figure VIIB in the online-only Data Supplement). These results indicate that functional RBPJ-binding motifs are required for the repression of Flk1in10 enhancer activity in venous endothelial cells. Although Rbpj forms an activation complex with NICD, it can also inhibit transcription through the binding of corepressors and recruitment of repression complexes in the absence of NICD.^31^ Because the expression of Notch receptors Notch1 and Notch4 is greatly enriched in the arterial endothelium, our results suggest that the low levels of Notch signaling in venous cells result in the formation of repressive complexes at the RBPJ motifs of the Flk1in10 enhancer. Supporting this, chromatin immunoprecipitation studies in mouse venous and arterial endothelial cells demonstrated that, although Rbpj can bind the Flk1in10 enhancer in both cell types, NICD is bound only in arterial endothelial cells (Figure VIIC in the online-only Data Supplement).

To further investigate the role of Rbpj/Notch in the regulation of Flk1in10, we examined the consequences of morpholino-induced *rbpj* knockdown in *tg*(*Flk1in10:GFP*) zebrafish (Figure 5G and 5H). In direct agreement with our transgene manipulation data, the depletion of Rbpj resulted in an expansion of Flk1in10:GFP expression into venous endothelial cells. This was also accompanied by a slight increase in GFP intensity, potentially a result of the increased cell proliferation and sprouting which occurs after Rbpj depletion.^32^ We next investigated the consequences of inhibiting NICD translocation to the nucleus by treating the *tg*(*Flk1in10:GFP*) embryos with the γ-secretase inhibitor N-[N-3,5-difluorophenacetyl]-l-alanyl-S-phenylglycine methyl ester (DAPM). Unlike the depletion of Rbpj, this chemical treatment was not expected to affect venous expression of Flk1in10 because Notch signaling is not active in the venous vasculature.^33^ Strikingly, DAPM treatment resulted in the compete ablation of Flk1in10:GFP expression at 72 hours post fertilization (Figure 5I), providing further evidence that Rbpj is able to repress Flk1in10 expression in the absence of Notch signaling. A schematic model summarizing these results is found in Figure VIII in the online-only Data Supplement.

### Potential Roles for Sox and Notch in Promoting Activity of Flk1in10 in Arterial Cells

Although our results suggest that only Ets and Gata factors transcriptionally activate Flk1in10 expression in endothelial cells, this may not preclude the involvement of other positive regulatory factors. All 3 characterized Flk1 enhancer elements contain essential ETS- and GATA-binding motifs, yet the activity of these enhancers overlaps only during the very early stages of vascular development. Because intact Notch signaling, essential for arterial differentiation, is itself downstream of VEGF through the Flk1 receptor,^8^ and loss of Notch signaling resulted in ablation of Flk1in10:GFP expression at 72 hours post fertilization, we wanted to investigate whether Rbpj/NICD binding could also provide a positive input to the Flk1in10 enhancer. The potential for such a role is supported by recent analysis of other arterial-restricted enhancers that have demonstrated activating roles for Rbpj/Notch and SoxF factors.^4,23^ The loss of Flk1in10 expression after DAPM-mediated sequestration of NICD suggested a possible positive role for Notch, yet these results may merely represent the acquisition of repression, not the loss of activation, and are not supported by the Rbpj morpholino results. However, loss of Rbpj in zebrafish resulted in a hyper-sprouting phenotype that may have also indirectly activated Gata-mediated Flk1in10 activity. Furthermore, analysis of mutations in the background of numerous ETS and GATA motifs created in multiple-copy transgenic mice and fish may inadvertently mask the minor effects of accessory transcriptional activators.

**Figure 6.**
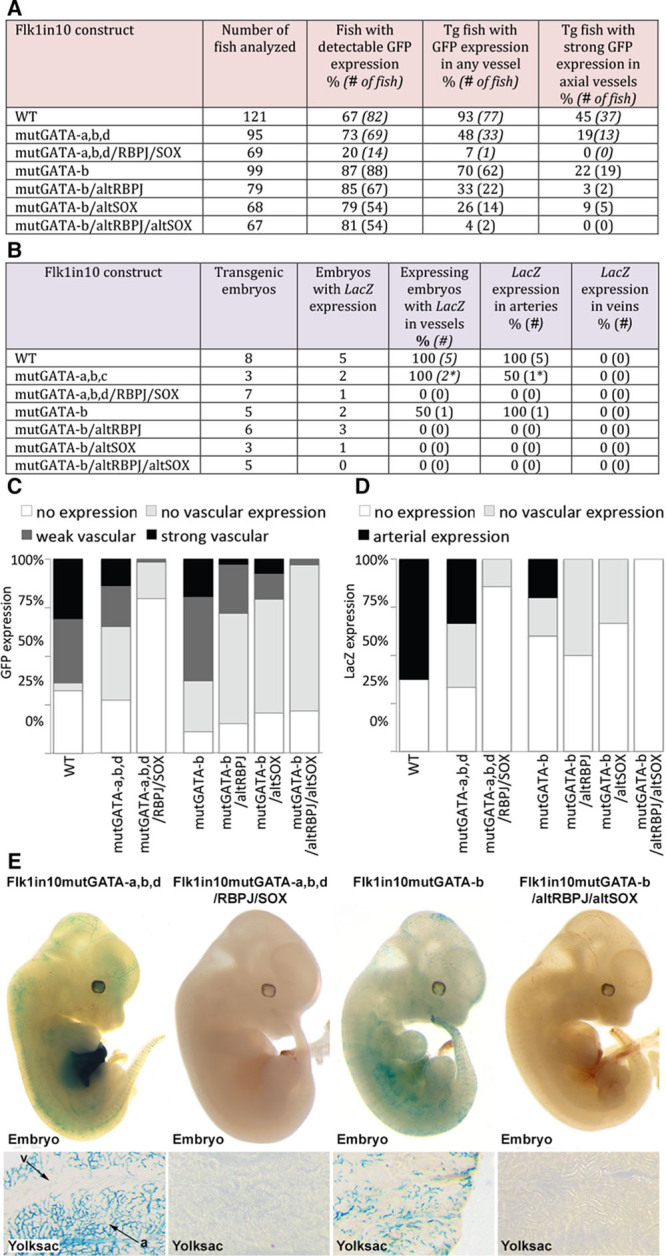
Ablation of RBPJ and SOX motifs alongside perturbation of GATA sites reveals a requirement for Rbpj/Notch and SoxF factors in the activation of Flk1in10 in arteries. **A and B**, Summary of reporter gene expression detected in (**A**) 48 h post fertilization (hpf) Tol2-mediated mosaic transient transgenic zebrafish embryos and (**B**) embryonic day (E) 12 transient transgenic mice. **C and D**, Bar charts representing zebrafish (**C**) and mouse (**D**) expression patterns of Flk1in10 constructs reported in **A** and **B**. **E**, Whole-mount embryos (**top**) and yolk sac tissues (**bottom**) of representative E12 X-gal–stained transient transgenic embryos expressing mouse Flk1in10mutGATA-a,b,d, mouse Flk1in10mutGATA-a,b,d/RBPJ/SOX, mouse Flk1in10mutGATA-b, and mouse Flk1in10mutGATA-b/altRBPJ/altSOX (**E**). a indicates artery; GFP, green fluorescent protein; and v, veins.

Consequently, we studied the potential positive role of Rbpj/NICD and SoxF on Flk1in10 by analyzing the effects of partial depletion of GATA in conjunction with RBPJ and SOX on Flk1in10 activity in transgenic zebrafish and mice. First, we generated a Flk1in10 construct in which one conserved GATA site (GATA-c) was left functional, whereas the other sites were mutated (mutGATAa,b,d). In both transgenic zebrafish and mice, the presence of a single functional GATA-c site resulted in increased enhancer activity in endothelial cells compared with mutGATA-all and mutGATA-b,c (Figure 6A–6E compared with Figure 4A and 4B). However, the expression levels were reduced compared with the WT Flk1in10 enhancer (Figure 6A and 6B). We further mutated this construct to also ablate the binding sites for SOX and RBPJ, creating Flk1in10mutGATA-a,b,d/RBPJ/SOX. Surprisingly, the additional mutation of the SOX and RPBJ sites resulted in a complete loss of reporter gene expression in both zebrafish and mouse transgenic embryos (Figure 6). These results suggest that, when the absolute binding levels of Gata are reduced, Rbpj/NICD and SoxF factors may play a transcriptionally activating role. Further transgenic analysis, this time using Flk1in10 constructs containing alternative mutations ablating either the GATA-b motif alone or in combination with SOX and RBPJ, resulted in similar patterns of transgene expression in both zebrafish and mouse models (Figure 6). These results therefore suggest that the RBPJ- and SOX-binding motifs, although not sufficient for enhancer activity alone, may provide the Flk1in10 enhancer with some level of positive regulatory input in arterial endothelial cells, alongside the more important role of Rbpj in the suppression of Flk1in10 in the vein.

## Discussion

In this study, we have identified a novel endothelial cell enhancer within the 10th intron of *Flk1*. Similar to other Flk1 regulatory elements, this enhancer requires both Ets- and Gata-binding motifs for transcriptional activity. However, Flk1in10 enhancer activity was restricted to the arterial compartment through both repressive and activating signals downstream of Rbpj/Notch and Sox regulatory pathways, demonstrating a direct link between the Notch and VEGF signaling pathways, and a novel regulatory role for Rbpj in repression of arterial identity in venous endothelial cells.

We have previously described an enhancer for the Notch ligand Dll4 (Dll4in3) which is also active in arterial endothelial cells.^4,34^ Although both enhancers can be described as directing arterial expression within the vasculature, they do not direct identical expression patterns: Dll4in3 is arterial specific throughout development, whereas Flk1in10 starts pan-vascular then gradually becomes arterially restricted. It is therefore unsurprising that the transcriptional mechanisms regulating these 2 enhancers are divergent. Rbpj plays no clear repressive role in Dll4in3 regulation, although Notch-dependent Rbpj activation in combination with SoxF factors is essential for enhancer activity. Conversely, our study demonstrates a role for Rbpj in repression of Flk1 expression in veins, in line with the view of Rbpj as a mediator of default repression in the absence of Notch signaling.^31^ These differences in expression outcome likely reflect the distinct upstream signals regulating the Notch and VEGF pathways in vascular development and further support the idea that neither the venous nor the arterial endothelial cell fully represents a default vascular state onto which the alternative identity is actively acquired.

Although Ets factors have long been known to be crucial for endothelial cell specification, recent studies have also implicated the Ets factor Erg in arterial specification downstream of VEGF.^34^ Because mutations to Ets-binding motifs in Flk1in10 resulted in loss of expression, it remains possible that Erg may contribute to the arterial-restricted activity of Flk1in10. However, the ETS-binding motifs were present and functional in the Flk1in10mutRBPJ/mutSOX enhancer, which was active in both arterial and venous endothelial cells, and the Flk1in10mutGATA/mutRBPJ/mutSOX enhancer, which was not active in endothelial cells at all. Consequently, although we cannot absolutely rule out an indirect role for Erg, it is clear that ETS motifs are not sufficient to achieve arterial specification or enhancer activation in the absence of other transcription factors.

Ablation of Gata2 specifically in endothelial cells in both mice and zebrafish does not recapitulate the Flk1 null phenotype, although later vascular differentiation was impaired.^27,35^ Although it is theoretically possible that the Flk1 locus could be independent of Gata factors via additional, undiscovered regulatory elements, this seems unlikely: all 3 known Flk1 enhancers require GATA-binding motifs to function, and expression of the orthologous *kdr* and *kdrl* genes was diminished in zebrafish after disruption of *gata2a*. Notably, Gata2 is not the only member of the Gata family expressed in the vasculature: both Gata3 and Gata6 are also expressed in endothelial cells during vascular development.^15^ Therefore, it is likely that additional members of the Gata transcription factor family can contribute to Flk1 regulation and at least partially compensate in the absence of Gata2.

Although it is known that VEGF and Notch both act in a conserved genetic pathway to promote arterial differentiation, and VEGF-mediated induction of Notch receptors requires intact Notch signaling, some models have suggested that Notch signaling can actively represses Flk1 levels. This was first proposed in studies of the angiogenic front in the postnatal retina, where decreased Notch levels in tip cells correspond with increased angiogenic sprouting and filopodia projections.^2^ However, the extent of direct Notch inhibition of Flk1 varies significantly in different experimental contexts, and although Flt4 (Vegfr3) expression inversely correlates with areas active in Notch signaling, the same is not true for Flk1.^16^ It is therefore likely that any repression of Flk1 expression downstream of active Notch during angiogenesis may reflect indirect repression via Flt4 instead of active Notch-mediated repression of Flk1 expression.^16,36^

Although enhancer studies primarily focus on the transcription factors essential for activity, it is likely that much of the crucial information governing vascular differentiation, patterning, and tissue specificity comes from nonessential, and consequently harder to assay, regulatory inputs. This research demonstrates that enhancer regulation of *Flk1* is more nuanced, and more complicated, than a simple on/off transcription factor switch, reflecting the reality of expression of an essential and multifaceted receptor within the heterogenous vasculature. Multiple enhancers, binding multiple transcription factors, permit different layers of transcriptional regulation and allow many different upstream cues to subtly alter expression levels. The arrangement of binding motifs within the Flk1in10 enhancer may enable Notch signaling to subtly suppress Flk1 expression in venous cells, whereas amplifying it in arterial cells, therefore providing a way to ensure correct arterial–venous differentiation without detrimentally influencing the other essential functions of the VEGF pathway in the vasculature.

## Acknowledgments

We thank N. Ahituv for providing Gateway-compatible vectors and M. Shipman for help with imaging.

## Sources of Funding

This work was supported by Ludwig Institute for Cancer Research Ltd and the Medical Research Council (MR/J007765/1) (N. Sacilotto, K. Liu, G. Bou-Gharios, and S. De Val) and the Wellcome Trust (090532/z/09/z) (C. Preece and B. Davies).

## Disclosures

None.

## Supplementary Material

## Highlights

- Novel endothelial enhancer for Flk1 located in the 10th intron.
- Flk1in10 enhancer expression becomes restricted to arterial endothelial cells during development.
- Notch pathway transcriptional effector RBPJ represses Flk1 enhancer expression in venous endothelial cells.

## References

[1] Jin SW, Patterson C (2009). The opening act: vasculogenesis and the origins of circulation.. Arterioscler Thromb Vasc Biol.

[2] Potente M, Gerhardt H, Carmeliet P (2011). Basic and therapeutic aspects of angiogenesis.. Cell.

[3] Lawson ND, Scheer N, Pham VN, Kim CH, Chitnis AB, Campos-Ortega JA, Weinstein BM (2001). Notch signaling is required for arterial-venous differentiation during embryonic vascular development.. Development.

[4] Sacilotto N, Monteiro R, Fritzsche M, Becker PW, Sanchez-Del-Campo L, Liu K, Pinheiro P, Ratnayaka I, Davies B, Goding CR, Patient R, Bou-Gharios G, De Val S (2013). Analysis of Dll4 regulation reveals a combinatorial role for Sox and Notch in arterial development.. Proc Natl Acad Sci USA.

[5] You LR, Lin FJ, Lee CT, DeMayo FJ, Tsai MJ, Tsai SY (2005). Suppression of Notch signalling by the COUP-TFII transcription factor regulates vein identity.. Nature.

[6] Hong CC, Peterson QP, Hong JY, Peterson RT (2006). Artery/vein specification is governed by opposing phosphatidylinositol-3 kinase and MAP kinase/ERK signaling.. Curr Biol.

[7] Claesson-Welsh L, Welsh M (2013). VEGFA and tumour angiogenesis.. J Intern Med.

[8] Lawson ND, Vogel AM, Weinstein BM (2002). Sonic hedgehog and vascular endothelial growth factor act upstream of the Notch pathway during arterial endothelial differentiation.. Dev Cell.

[9] Shalaby F, Rossant J, Yamaguchi TP, Gertsenstein M, Wu XF, Breitman ML, Schuh AC (1995). Failure of blood-island formation and vasculogenesis in Flk-1-deficient mice.. Nature.

[10] Bussmann J, Lawson N, Zon L, Schulte-Merker S, Zebrafish Nomenclature Committee (2008). Zebrafish VEGF receptors: a guideline to nomenclature.. PLoS Genet.

[11] Covassin LD, Villefranc JA, Kacergis MC, Weinstein BM, Lawson ND (2006). Distinct genetic interactions between multiple Vegf receptors are required for development of different blood vessel types in zebrafish.. Proc Natl Acad Sci USA.

[12] Kappel A, Rönicke V, Damert A, Flamme I, Risau W, Breier G (1999). Identification of vascular endothelial growth factor (VEGF) receptor-2 (Flk-1) promoter/enhancer sequences sufficient for angioblast and endothelial cell-specific transcription in transgenic mice.. Blood.

[13] Ema M, Takahashi S, Rossant J (2006). Deletion of the selection cassette, but not cis-acting elements, in targeted Flk1-lacZ allele reveals Flk1 expression in multipotent mesodermal progenitors.. Blood.

[14] Ishitobi H, Wakamatsu A, Liu F, Azami T, Hamada M, Matsumoto K, Kataoka H, Kobayashi M, Choi K, Nishikawa S, Takahashi S, Ema M (2011). Molecular basis for Flk1 expression in hemato-cardiovascular progenitors in the mouse.. Development.

[15] Val SD (2011). Key transcriptional regulators of early vascular development.. Arterioscler Thromb Vasc Biol.

[16] Benedito R, Rocha SF, Woeste M, Zamykal M, Radtke F, Casanovas O, Duarte A, Pytowski B, Adams RH (2012). Notch-dependent VEGFR3 upregulation allows angiogenesis without VEGF-VEGFR2 signalling.. Nature.

[17] Dejana E (2010). The role of wnt signaling in physiological and pathological angiogenesis.. Circ Res.

[18] Goumans MJ, Liu Z, ten Dijke P (2009). TGF-beta signaling in vascular biology and dysfunction.. Cell Res.

[19] Kent WJ, Sugnet CW, Furey TS, Roskin KM, Pringle TH, Zahler AM, Haussler D (2002). The human genome browser at UCSC.. Genome Res.

[20] Birnbaum RY, Everman DB, Murphy KK, Gurrieri F, Schwartz CE, Ahituv N (2012). Functional characterization of tissue-specific enhancers in the DLX5/6 locus.. Hum Mol Genet.

[21] Chi NC, Shaw RM, De Val S, Kang G, Jan LY, Black BL, Stainier DY (2008). Foxn4 directly regulates tbx2b expression and atrioventricular canal formation.. Genes Dev.

[22] Kieffer-Kwon KR, Tang Z, Mathe E (2013). Interactome maps of mouse gene regulatory domains reveal basic principles of transcriptional regulation.. Cell.

[23] Robinson AS, Materna SC, Barnes RM, De Val S, Xu SM, Black BL (2014). An arterial-specific enhancer of the human endothelin converting enzyme 1 (ECE1) gene is synergistically activated by Sox17, FoxC2, and Etv2.. Dev Biol.

[24] Thompson JD, Higgins DG, Gibson TJ (1994). CLUSTAL W: improving the sensitivity of progressive multiple sequence alignment through sequence weighting, position-specific gap penalties and weight matrix choice.. Nucleic Acids Res.

[25] De Val S, Black BL (2009). Transcriptional control of endothelial cell development.. Dev Cell.

[26] Lugus JJ, Chung YS, Mills JC, Kim SI, Grass J, Kyba M, Doherty JM, Bresnick EH, Choi K (2007). GATA2 functions at multiple steps in hemangioblast development and differentiation.. Development.

[27] Zhu C, Smith T, McNulty J (2011). Evaluation and application of modularly assembled zinc-finger nucleases in zebrafish.. Development.

[28] Khandekar M, Brandt W, Zhou Y, Dagenais S, Glover TW, Suzuki N, Shimizu R, Yamamoto M, Lim KC, Engel JD (2007). A Gata2 intronic enhancer confers its pan-endothelia-specific regulation.. Development.

[29] Randi AM, Sperone A, Dryden NH, Birdsey GM (2009). Regulation of angiogenesis by ETS transcription factors.. Biochem Soc Trans.

[30] Francois M, Koopman P, Beltrame M (2010). SoxF genes: key players in the development of the cardio-vascular system.. Int J Biochem Cell Biol.

[31] Bray SJ (2006). Notch signalling: a simple pathway becomes complex.. Nat Rev Mol Cell Biol.

[32] Siekmann AF, Lawson ND (2007). Notch signalling limits angiogenic cell behaviour in developing zebrafish arteries.. Nature.

[33] Lin FJ, Tsai MJ, Tsai SY (2007). Artery and vein formation: a tug of war between different forces.. EMBO Rep.

[34] Wythe JD, Dang LT, Devine WP, Boudreau E, Artap ST, He D, Schachterle W, Stainier DY, Oettgen P, Black BL, Bruneau BG, Fish JE (2013). ETS factors regulate Vegf-dependent arterial specification.. Dev Cell.

[35] Lim KC, Hosoya T, Brandt W, Ku CJ, Hosoya-Ohmura S, Camper SA, Yamamoto M, Engel JD (2012). Conditional Gata2 inactivation results in HSC loss and lymphatic mispatterning.. J Clin Invest.

[36] Zarkada G, Heinolainen K, Makinen T, Kubota Y, Alitalo K (2015). VEGFR3 does not sustain retinal angiogenesis without VEGFR2.. Proc Natl Acad Sci USA.

