# Supplementary material for: An Intronic Flk1 Enhancer Directs Arterial-Specific Expression via RBPJ-Mediated Venous Repression
Source: Arterioscler Thromb Vasc Biol. 2016 May 25;36(6):1209–19. doi: 10.1161/ATVBAHA.116.307517 (PMC4894770; doi:10.1161/ATVBAHA.116.307517)
Supplement: Supplementary file 1 [file atv-36-1209-s001.pdf]

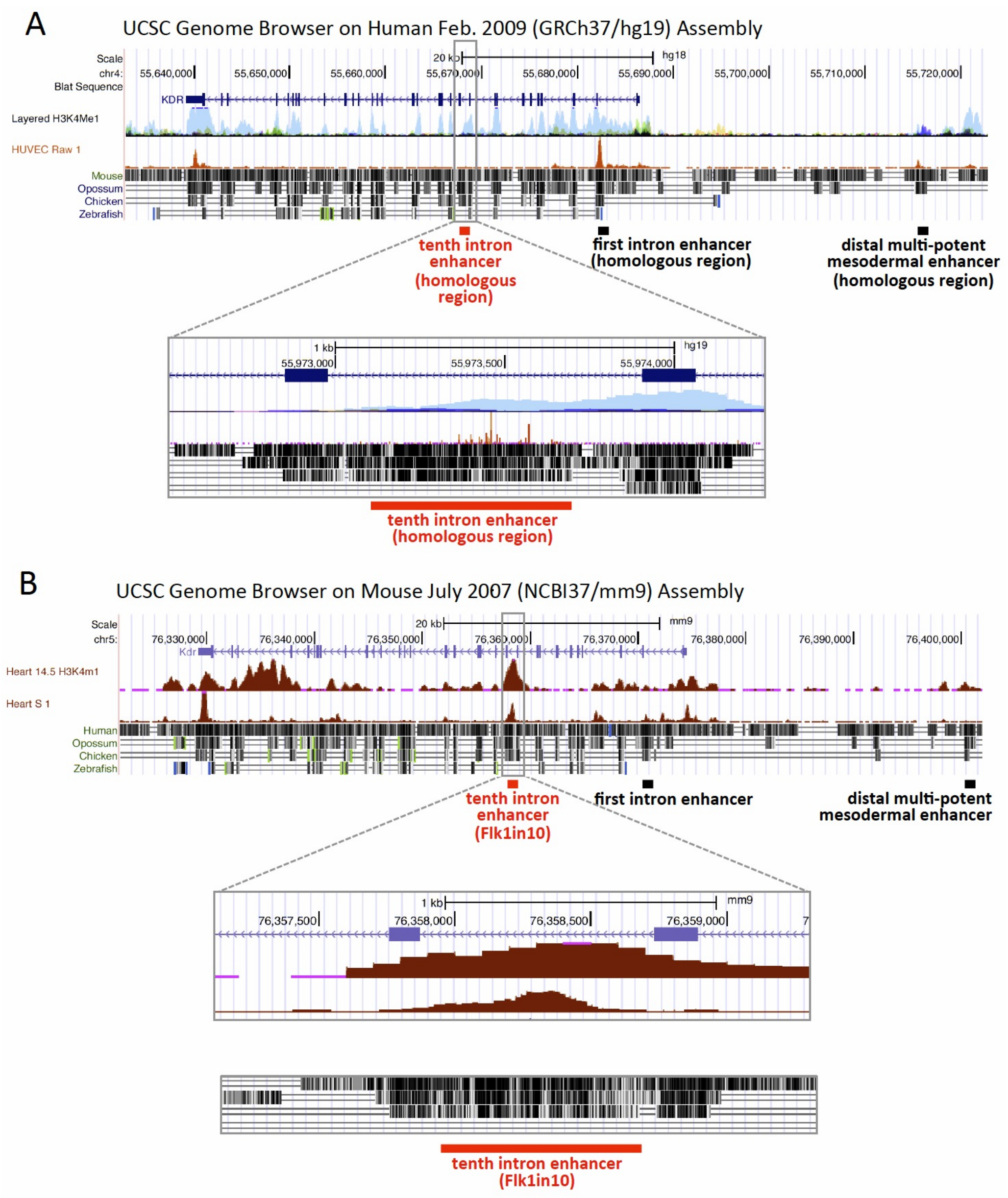

Supplemental Figure I.

A. Schematic representation of the human *KDR* locus from UCSC ENCODE Browser. This information was used to identify the intron 10 region as a putative enhancer. HUVEC specific H3K4Me1 peaks in light blue, HUVEC specific DNase I HS peaks in red, sequence conservation between human and mouse, opossum, chicken and zebrafish indicated by vertical black lines. Region homologous to mouse Flk1in10 enhancer indicated with red bar, regions homologous to mouse Flk1 intron 1 enhancer and DMME enhancers marked with black horizontal bars.

B. Schematic representation of the mouse *Flk1* locus from UCSC ENCODE Browser. E14.5 heart specific H3K4Me1 upper peaks in maroon, heart specific DNase I HS lower peaks in maroon, sequence conservation between mouse and human, opossum, chicken and zebrafish indicated by vertical black lines. Region encompassing the Flk1in10 enhancer indicated with red bar, regions encompassing Flk1 intron 1 enhancer and DMME enhancers marked with black horizontal bars. Note that other untested intronic regions also have enhancer-associated marks indicative of putative enhancer elements. Genome browser screen shots from http://genome.ucsc.edu [19].

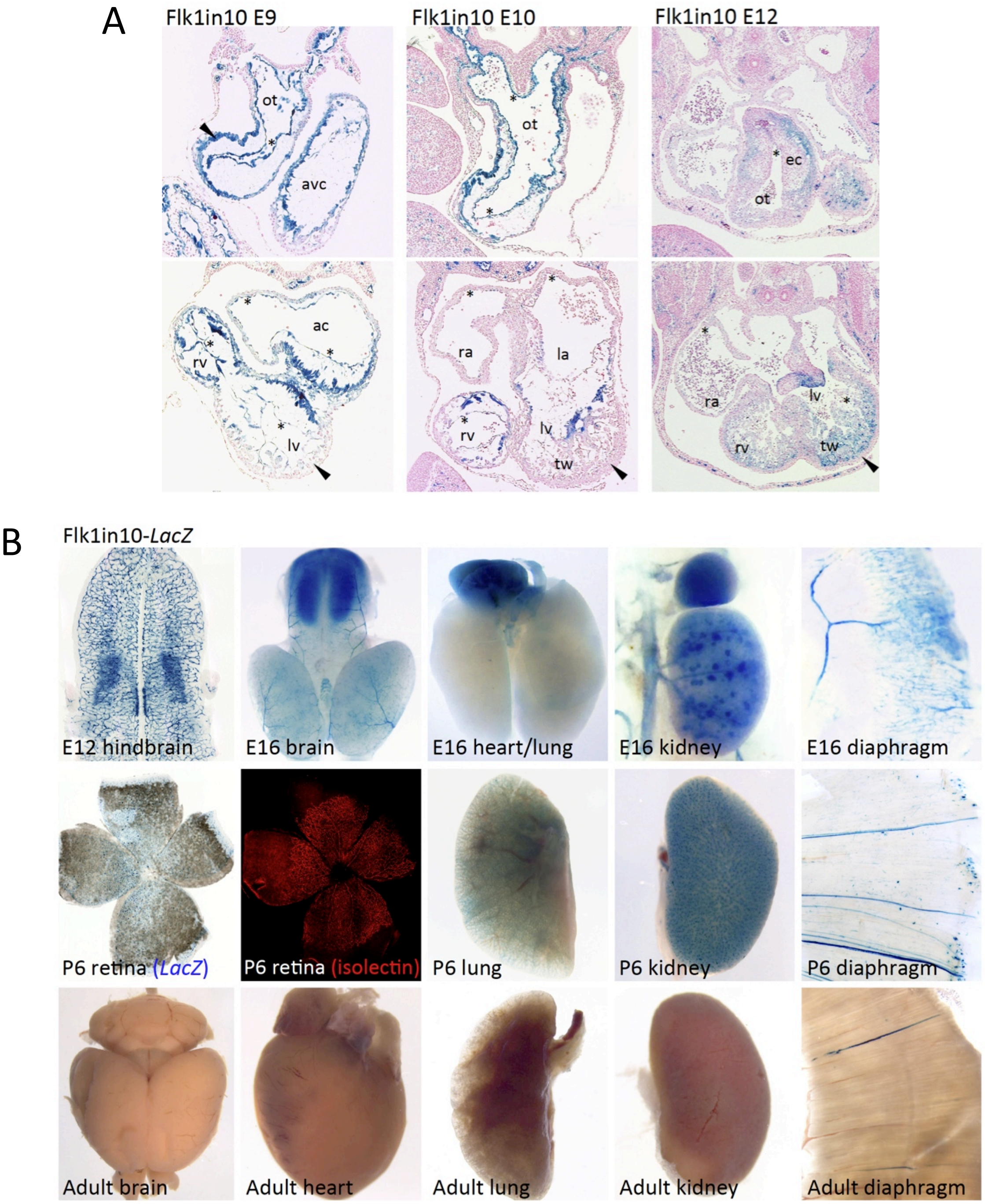

Supplemental Figure II.

A. Transverse sections through different levels of the heart of E9, E10 and E12 Flk1in10-*LacZ* transgenic mouse embryos. * indicates endocardium, arrowhead indicates myocardium, ac common atrial canal, avc atrial-ventricular canal, ec endocardial cushion, la left atria, lv left ventricle, ra right atria, rv right ventricle, ot outflow tract, tw trabecular wall.

B. Time-course of organs from Flk1in10-*LacZ* transgenic mice embryos, P6 pups and 10 week old adult mice. All organs were dissected and stained separately for X-gal, with exception of P6 retina (isolectin) picture, in which the P6 retina depicted to the right was stained for expression of the pan-endothelial marker isolectin b4 conjugated to DyLight 594 (red).

A

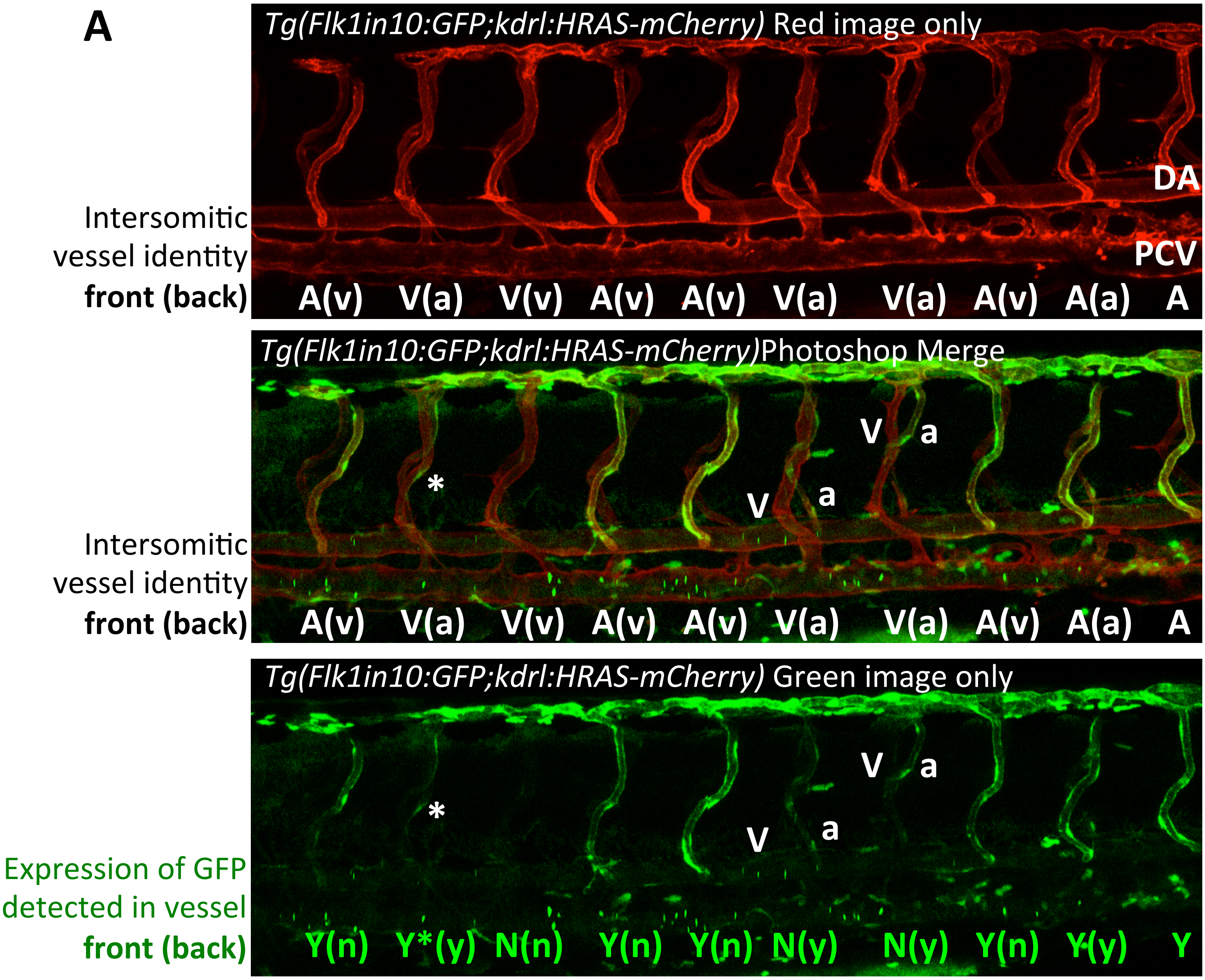

B

**Identity score**	:	A	V	V	A	A	V	V	A	A	A
**GFP score**	:	Y	Y	N	Y	Y	N	N	Y	Y	Y

Supplemental Figure III.
A. Detailed example of the analysis of arterial:venous identity of intersegmental sprouts used in Figure 2. Pictures denote single image of a representative 72 hpf *tg(Flk1in10:GFP;kdrl:HRAS-mCherry)* embryo. Top panel denotes mCherry image only, in which the pan-vascular kdrl:HRAS-mCherry transgene is expressed in all intersegmental vessels as well as the dorsal aorta (DA) and posterior cardinal vein (PCV). Using this image we were able to assess the identity of each intersegmental vessel by whether it connects to the DA (arterial) or PCV (venous). The transparency of the embryo meant that we image two intersegmental vessels for each segment, one in the foreground denoted by UPPERCASE and the other in the background, denoted by bracketed lowercase. The middle image denotes both GFP and mCherry expression, and the bottom image just GFP expression. For each intersegmental vessel detected by kdrl:HRAS-mCherry expression, we scored whether GFP expression was detected (Y) or not (N). Vessels in foreground recorded as UPPERCASE, vessels in background as bracketed lowercase. * represents GFP in one cell, still recorded as GFP expression (Y) by our method.
B. Creation of identity and GFP score used to create the bar chart in Figure 2. Records only foreground intersegmental identity and GFP expression.

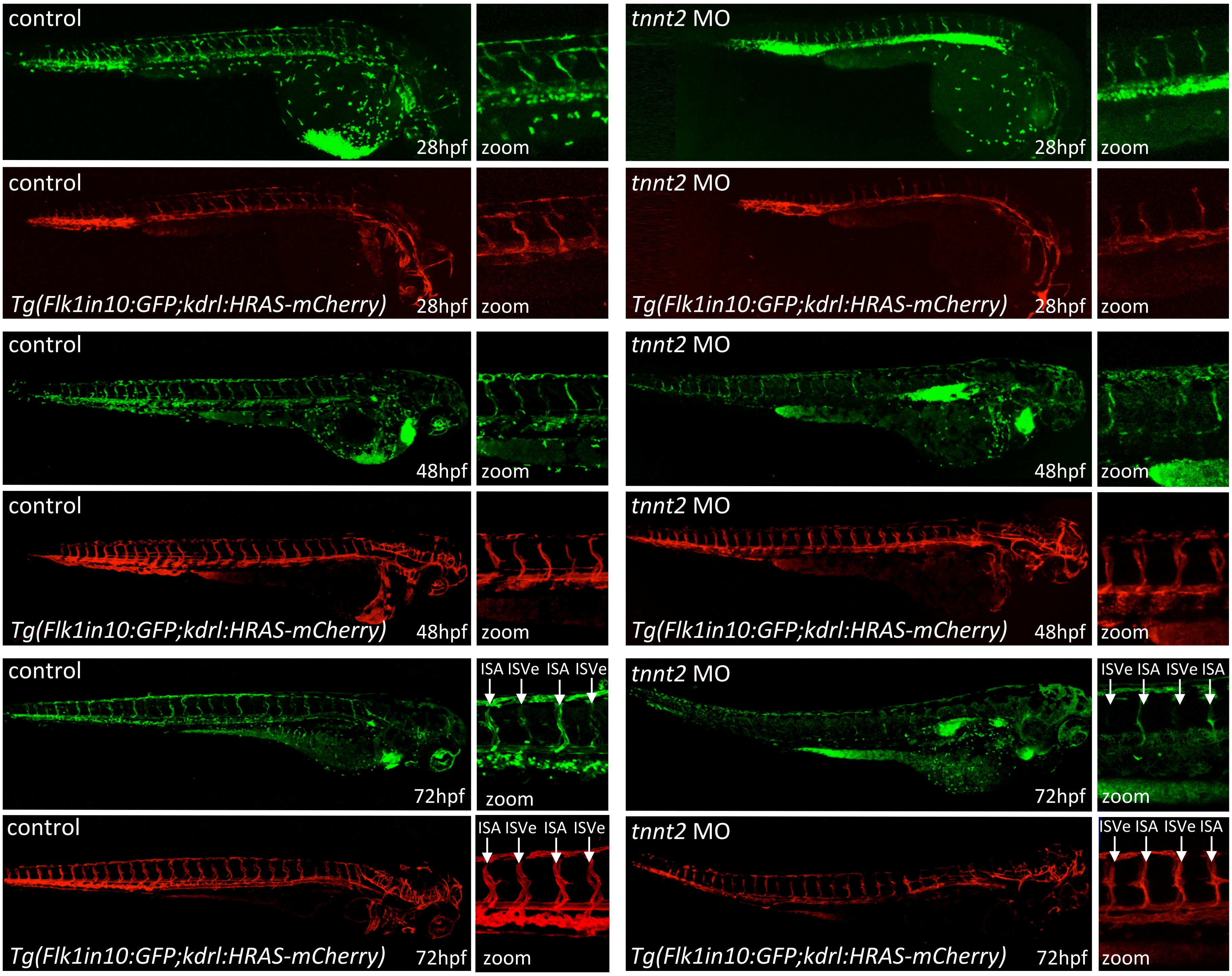

Supplemental Figure IV.
Loss of circulation does not prevent arterial restriction of Flk1in10:GFP expression. Analysis of the effect of 4ng *tnnt2* MO in 28, 48 and 72 hpf *tg(Flk1in10:GFP;kdrl:HRAS-mCherry)* embryos. kdrl:HRAS-mCherry marks all vessels. ISA intersegmental artery, ISVe intersegmental vein.

Flk1in1-*LacZ* E12

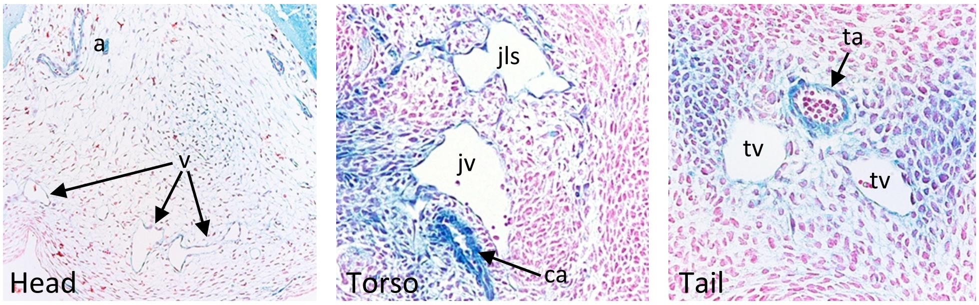

Supplemental Figure V

Transverse sections through Flk1 intron1-LacZ transgenic embryo at E12 demonstrates transgene expression in both venous and arterial endothelial cells. A artery, ca carotid artery, jls jugular lymph sac, jv jugular vein, ta tail artery, tv tail vein, v vein.

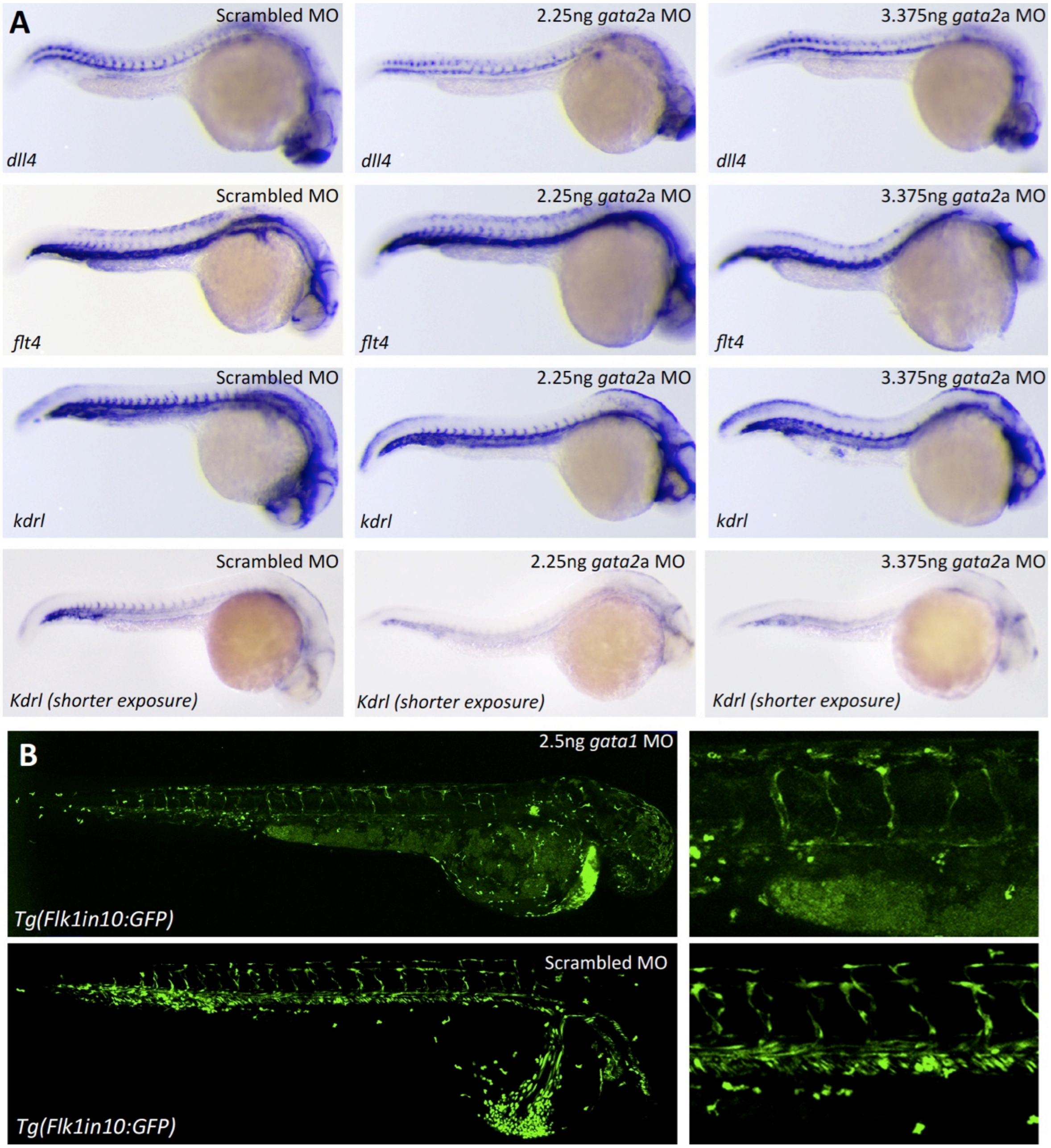

Supplemental Figure VI.

A. Analysis of scrambled, 2.25ng and 3.375 ng *gata2a* MO in 26 hpf WT zebrafish embryos, using whole-mount *in situ* hybridization with probes against arterial marker *dll4*, venous marker *flt4* and *kdrl*.

B. Analysis of 2.5ng *gata1* MO in 36 hpf *tg(Flk1in10:GFP)* embryos.

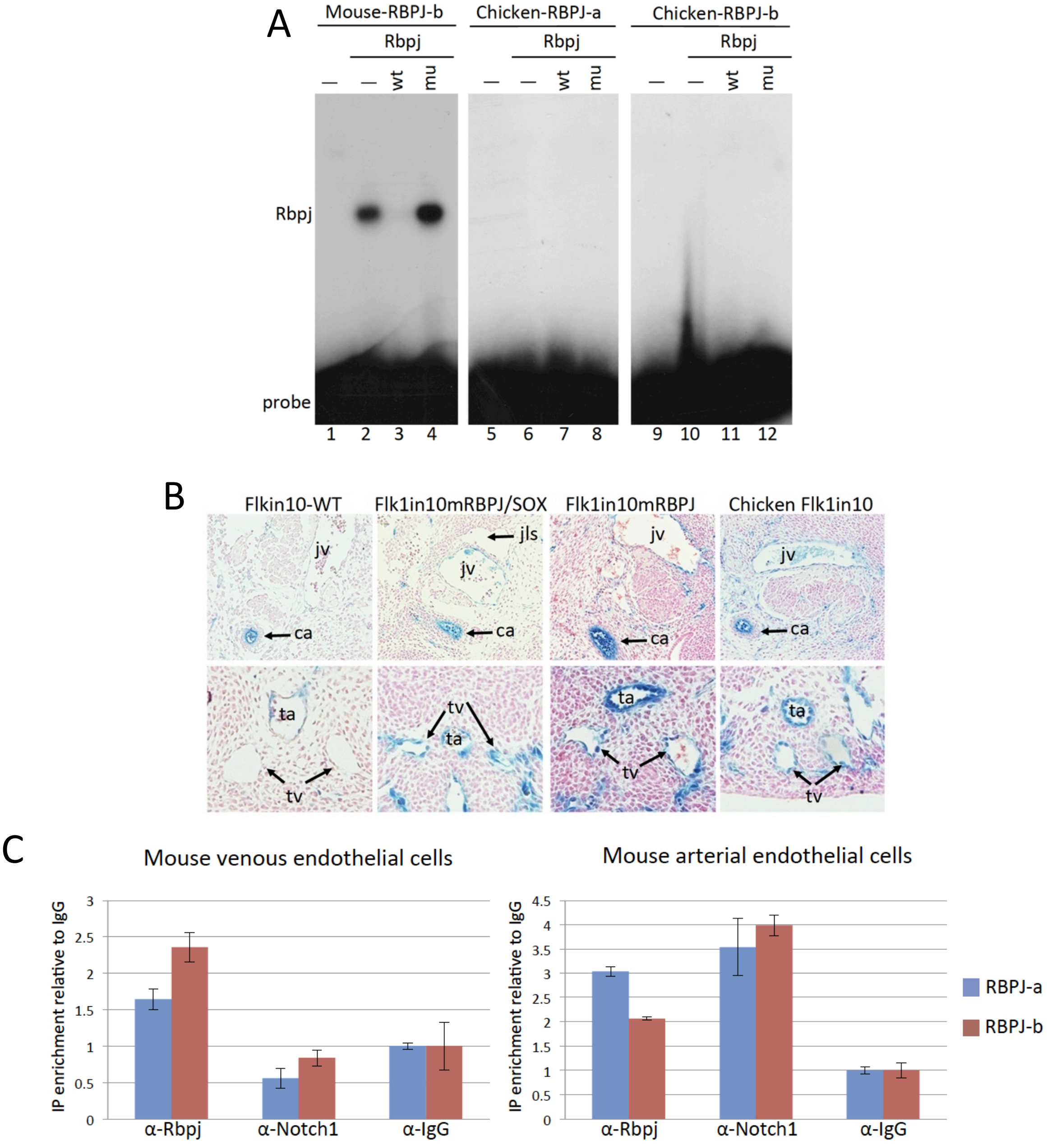

Supplemental Figure VII.

A. Radiolabeled oligonucleotide probe encompassing mouse Flk1in10 RBPJ-a (lanes 1-4) was able to robustly bind to recombinant Rbpj protein (lane 2), but the orthologous chicken Flk1in10 RBPJ-a (lanes 5-8) and chicken Flk1in10 RBPJ-b (lanes 9-12) motifs were unable to bind in the same experimental conditions (lane 6 and 10). Rbpj protein was competed by excel unlabeled self-probe (lane 3) but not by mutant self-probe (4). Experiment was done in parallel, n=2.

B. Transverse sections from representative E12 X-gal stained transient transgenic embryos expressing mouse Flk1in10-WT, mouse Flk1in10mRBPJ/SOX, mouse Flk1in10mRBPJ and chicken Flk1in10 WT. Upper line shows carotid artery and jugular vein, lower line shows tail artery and paired veins. Ca carotid artery, jls jugular lymph sac, jv jugular vein, ta tail artery, tv tail vein.

C. Chromatin immunoprecipitation assays were performed with anti-Rbpj, anti-Notch1 and control immunoglobulin G (IgG) antibodies. The DNA content of the immunoprecipitates was analyzed by real-time PCR for Flk1in10. Results were normalized by input and IP enrichment expressed relative to the IgG. Error bars mean standard deviation of n=2.

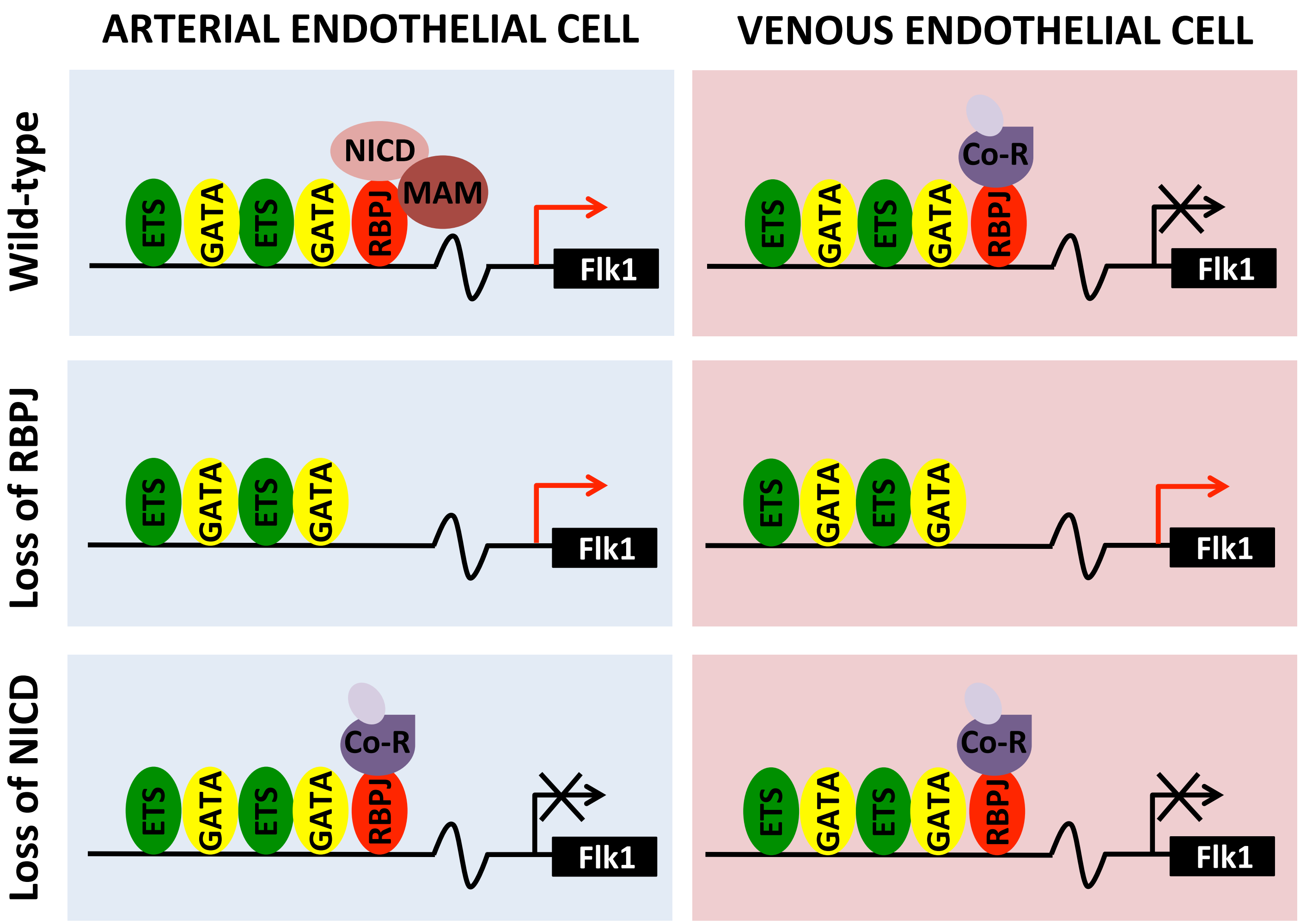

Supplemental Figure VIII.

Schematic summarising the effects that perturbations to the Notch pathway have on the transcriptional activation of the Flk1in10 enhancer. NICD denotes Notch intracellular domain, MAM denotes Mastermind-like transcriptional co-activator, Co-R denotes co-repressors [30]. Red angled arrow indicates transcriptional activation, black arrow with strike-through indicates transcriptional repression.
