# Supplementary material for: An Intronic Flk1 Enhancer Directs Arterial-Specific Expression via RBPJ-Mediated Venous Repression
Source: Arterioscler Thromb Vasc Biol. 2016 May 25;36(6):1209–19. doi: 10.1161/ATVBAHA.116.307517 (PMC4894770; doi:10.1161/ATVBAHA.116.307517)
Supplement: Supplementary file 3 [file atv-36-1209-s003.pdf]

Cloning of Flk1in10 WT and mutant enhancers constructs

The 826 bp Flk1in10 enhancer was generated by PCR from mouse genomic DNA using Flk1in10 mouse F and R primers (primer sequence provide in table below). The orthologous 1012 bp chicken Flk1in10 enhancer was generated by PCR from chicken genomic DNA using the Flk1in10 chicken F and R primers (primer sequence provide in table below). PCR products were cloned into the pCR8 vector using the pCR8/GW/TOPO TA Cloning Kit (Invitrogen, K2500-20) following manufacturer's instructions. The Flk1 intron 1 enhancer was generated by PCR from mouse genomic DNA using Flk1in1 mouse F and R primers and cloned in pCR8 as described.

Mutant versions of the mouse Flk1in10 enhancer were initially generated as custom-made, double-stranded linear DNA fragments (GeneArt® Strings™, Life Technologies) and were subsequently treated as PCR products described above. The sequences of each mutant enhancer DNA fragment are provided in the DNA fragment sequence information. Once cloning was confirmed, the enhancer sequence was transferred from the pCR8/GW/enhancer entry vector to a suitable destination vector using Gateway LR Clonase II Enzyme mix (Life Technologies, 11791-100) following manufacturer's instructions. For mouse transgenesis, the enhancer was cloned into the hsp68-*LacZ*-Gateway vector (provided by N. Ahituv). For zebrafish transgenesis, the enhancer was cloned into the E1b-GFP-Tol2 vector (provided by N. Ahituv).

Generation and analysis of transgenic fish

F0 transient mosaic transgenic zebrafish embryos were generated by the Tol2 system (1). Briefly, 0.5 nl of 50 ng/µl Tol2 transposase capped mRNA (mMESSAGE mMACHINE® SP6 Kit, Ambion) and 60 ng/µl pE1b/enhancer/GFP expression vector were injected into 1-cell embryos obtained by natural spawning of wild type (WT) adult zebrafish raised and maintained at 28.5°C in system water. Embryos were maintained in E3 medium (5 mM NaCl; 0.17 mM KCl; 0.33 mM $CaCl_2$; 0.33 mM $MgSO_4$) at 28.5 °C.

The Flk1in10:GFP stable line *tg(Flk1in10:GFP*) was generated from an initial outcross of adult F0 carriers generated by the Tol2 system described above. GFP positive embryos from these initial outcrosses (F1 generation) were analyzed for expression, and two representative lines selected. The *tg(Flk1in10:GFP)* line was intercrossed with *tg(kdrl:HRAS-mCherry)*[1] to enable visualization of the entire vasculature.

To image, all embryos were dechorionated and anesthetized with 0.1% tricaine mesylate. For analysis of transient transgenic zebrafish, single embryos were transferred into a flat bottom 96-well plate plate, and GFP reporter gene expression screened with a Zeiss LSM 710 confocal microscope at 46-50 hpf. The total number of injected fish, the total number of fish with any detectable GFP expression, and the total number of fish with any detectable GFP expression in the vasculature were all noted. Whole fish were imaged using the "tile scan" command, combined with Z-stack collection under a confocal microscope Zeiss LSM 710 MP (Carl Zeiss) at 488nm excitation and 509nm emission (EGFP) and 587 nm and 610 nm (mCherry), respectively. Movies were generated by collecting images at 24FPS under a confocal microscope Zeiss LSM 710 MP (Carl Zeiss) using the "time series" command.

To assess GFP expression patterns in 72 hpf *tg(Flk1in10:GFP; kdrl:HRAS-mCherry)* zebrafish embryos, still images were taken and assessed. The identity of

the intersegmental vessel was established using the expression of the pan-vascular kdrl:HRAS-mCherry reporter [1] and identifying whether the vessel ran into the dorsal aorta (intersegmental artery) or cardinal vein (intersegmental vein). Each vessel was then assessed for GFP expression.

Generation and analysis of transgenic mice

All animal procedures were approved by local ethical review and licensed by the UK Home Office. Transgenic mice were generated by oocyte microinjection as described previously [2].

Transgenic mouse embryos were collected along with yolk sac and placenta. After dissection, samples were rinsed in ice-cold 1 X PBS and fixed in 2% PFA, 0.2% glutaraldehyde, 1 X PBS at 4°C as indicated below:

Age	Fixation time	Further processing
E7.5-8.5	10 min	Embryos were rinsed twice in 1x PBS, then incubated in PBS for 30 min at 4 °C
E8.5-9.0 / hindbrain	20 min	
E9.5	30 min	
E10-11.5	60 min	
E12.5	1.5 h	Embryos were rinsed twice in PBS, than incubated 2 x 30 min in rinse solution (0.1% sodium deoxycholate, 0.2% Nonidet P-40, 2 mM MgCl2, 1 X PBS) at 4 °C
E13.5 / organs	2 h	
E14.5	2.5 h	
E15.5	3 h	
E16.5-17.5	4 h	
Retinas were fixed in 4% PFA for 1h on ice		

After fixation and rinse, embryos were stained overnight at room temperature in 1 mg/ml 5-bromo-4-chloro-3-indolyo-β-D-galactoside solution (X-gal) containing 5 mM potassium ferrocyanide, 5 mM ferricyanide, 0.1% sodium deoxycholate, 0.2% Nonidet P-40, 2 mM $MgCl_2$ and 1 X PBS. After staining, embryos were rinsed through a series of 1 X PBS washes, then fixed overnight in 4% paraformaldehyde at 4 °C.

Imaging of whole embryos and organs was performed using a stereo microscope (Leica M165C) equipped with a ProGres CF Scan camera (Jenoptik) and ProgRes CapturePro software (Jenoptik). For each enhancer, embryos were also sectioned for histological analysis to investigate X-gal staining patterns. For histological analysis, embryos were dehydrated through a series of ethanol washes, cleared by xylene and paraffin wax-embedded. 5 or 6-μm sections were prepared, de-waxed, and counterstained with nuclear fast red (Electron Microscopy Sciences). Co-staining for Endomucin and β-galactosidase was performed on de-waxed sections using a dilution of 1:100 rat anti-Endomucin antibody (Santa Cruz, sc-65495) and 1:100 mouse anti- β-galactosidase (MP Biomedicals, 559761) in PBS as described previously [3].

Retinas and hindbrains were incubated in blocking buffer (0.5% Tween + 1% Normal Horse Serum in PBS) for 1h and stained with DyLight 594 GSL I – B4 Lectin (Vector, DL-1207) in PBS+ 0.5% Tween for 2h followed by 5 x 10 min washes in PBS + 0.5% Tween. Retinas and hindbrains were then flat mounted using VECTASHIELD mounting medium (Vector, H-1200) and imaged using imaged using the “tile scan” command, combined with Z-stack collection under a confocal

microscope Zeiss LSM 710 MP (Carl Zeiss) at 590nm excitation and 617nm emission.

The placenta was used for genotyping. Tissue samples were incubated overnight at 55°C with 500 µL GNT buffer (50mmol/L KCl, 1.5mmol/L $MgCl_2$, 10mmol/L Tris-pH8, 0.01% gelatin, 0.45% nonidet P40, 0.45% Tween) and proteinase K (10mg/ml). Afterwards, the solution was heated to 95°C for 30 minutes and centrifuged for 1 minute at maximum rpm in a benchtop centrifuge. 0.5 µL of this supernatant were subsequently used in a PCR reaction with the GoTag Green master mix (Promega, M7122) using LacZ PCR primer F and R (sequence provide in table below).

Morpholinos and Chemical Treatments.

Antisense morpholino oligonucleotides were as described previously (Gata1 ATG MO and gata2a splice MO[4]; Sox7 ATG MO and SOX18 ATG MO[5]; RbpJ ORF MO [1,6], tnnt2 MO[2,7]). MOs were injected into 1- to 2-cell wild-type or *tg*(Flk1in10:GFP) embryos, at a concentration depicted in the figure legends (maximum injection volume of 2 nl). For inhibition of Notch signaling embryos were incubated with 100 µM DAPM (Calbiochem) starting at 26 hpf.

Zebrafish whole mount in situs

In situ hybridization procedures used the following probes: *flt4*[4,8], *dll4*[5,9], *kdrl* [10]. *kdr* probe was generated as a DNA fragment (sequence listed below) from GeneArt® Strings™ (Life Technologies), cloned using the pCR2/TOPO/TA cloning kit (Invitrogen 450641) and transcribed using SP6 and T7. *In situ* analysis was conducted as previously described[11]. Briefly, embryos were collected at 26hpf and fixed overnight at 4 °C in 4% paraformaldehyde. Fixed embryos were dehydrated and stored at -20 °C in 100% ethanol. When needed embryos were rehydrated in PBST, treated with 10µg/ml proteinase K (Sigma) for 10 minutes, followed by two PBST washes. Thereafter, the embryos were fixed with 4% PFA for 20 min and washed five times with PBST. Embryos were transferred into hybridization solution (50% formamide, 5 × SSC, 0.1% Tween 20, 50 µg/ml heparin, 500 µg/ml of tRNA adjusted, 10 mM citric acid) for 2 hours at 65 °C, then transferred into diluted antisense riboprobe/hybridization solution and incubated overnight at 65 °C. Probes were removed and embryos transferred to a Biolane HT-1 *in situ* machine (Intavis). Embryos were washed through a dilution series into 2 x SSC followed by 0.2 x SSC at 65 °C. The embryos were then taken through room temperature dilution washes into 100 % MABT (0.1M Maleic Acid, 0.15 M NaCl, pH 7.5). Non-specific sites were blocked with MAB block (MABT with 2% Boehringer block reagent) at room temperature and incubated for 15 hours with anti-DIG antibody (Roche) at 1:2000 at 4 °C, before washing in MABT. Prior to staining, embryos were washed in AP buffer and the *in situ* signal developed at room temperature with BM Purple (Roche). Staining was stopped as appropriate by fixation in 4% paraformaldehyde. Embryos were transferred to 80% glycerol for imaging and storage.

Electrophoretic mobility shift assay

Electrophoretic mobility shift assay (EMSAs) were performed as described previously [3]. Proteins were made using the TNT Quick Coupled Transcription/Translation system as described in the manufacturer's directions. The full length Sox7 and Gata2 were in the pCITE2 plasmid, and transcribed using T7 polymerase. Etv2 was in the pCS2 plasmid, and transcribed using Sp6 polymerase.

Foxc2 was in the pCR2.1 plasmid and transcribed using T7, Rbpj was in the pcDNA3.1 plasmid and transcribed using T7.

To label the probe, double stranded oligonucleotides were labeled with 32P-dCTP, using Klenow (Promega) to fill in overhanging 5' ends, and purified on a non-denaturing polyacrylamide-TBE gel. 20 µl binding reactions consisted of 3-5 µl protein or lysate control and 2 µl 10X binding buffer (40mM KCl, 15 mM HEPES pH 7.9, 1 mM EDTA, 0.5 mM DTT, 5% glycerol). For Etv2 and Rbpj, 0.5 µg of poly dI-dC was used. For Foxc2, 0.25µg of poly-dI-dC was used, and for Sox7, 0.25µg poly-dG-dC was used. For competitor lanes, a 100-fold excess of competitor DNA was added in a volume of 1µl. Binding reactions were incubated at room temperature for 20 minutes before the addition of radiolabeled probed, after which they were incubated an additional 20-40 minutes. Gels were electrophoresed on a 6% non-denaturing polyacrylamide gel.

Chromatin immuoprecipitation (ChIP)

Mouse aortic and venous endothelial cells (C57BL/6 mouse primary vein endothelial cells C57-6009 and mouse primary aortic endothelial cells C57-6052, CellBiologics) were grown to 70% confluency in Endothelial Cell Medium (CellBiologics M1168), and fixed in 1% methanol-free PFA at 4C for 10 min. Cross-linked cells were washed twice with ice-cold PBS, carefully lysed in cell-lysis buffer supplemented with protease inhibitors (Sigma) to obtain intact nuclei. Nuclei were subsequently sedimented at 1000 x g for 5 min at 4C and lysed in nuclei lysis buffer (50mM Tris pH8.1, 10mM EDTA, 1% SDS). Chromatin was sonicated to obtain fragments of an average size of 400 bp using a Covaris sonicator. Diluted and pre-cleared chromatin was then immunoprecipitated wih RbpJ, Notch1 and IgG antibodies[3]. The ChIP was analysed by qPCR using primers described below in table. Results were normalized by input and IP enrichment expressed relative to the IgG. Error bars mean standard deviation of n=2.

PCR, EMSA and Morpholino primers

Lowercase indicate non-homologous 5' tag to permit radioisotope labeling. *Lowercase italics* indicate mutated nucleotides.

**Oligo name**	**Oligo sequence**
LacZ PCR primer F	5'-GTTGCAGTGCACGGCAGATACACTTGCTGA-3'
LacZ PCR primer R	5'-GCCACTGGTGTGGGCCATAATTCAATTCGC-3'
Flk1in10 mouse F	5'-GCATGTCAAGATTTGACTTC-3'
Flk1in10 mouse R	5'-CACGATGGCAAGTGAAACAG—3'
Flk1in10 chicken F	5'-GCTGATCAACAAATCAGATG-3'
Flk1in10 chicken R	5'-AGCACATATGGCATAGGGAG-3'
Flk1in10 GATA-a F	ctagGTCCTGAGGATACAGGAGGG
Flk1in10 GATA-a mut_F	ctagGTCCTGAGG*gg*ACAGGAGGG
Flk1in10 GATA-b_F	ctagTAGACCTTGATAAGCCTGGG
Flk1in10 GATA-b mut_F	ctagTAGACCTTG*gg*AAGCCTGGGC
Flk1in10 GATA-c_F	ctagGGGCTAGGTTTATCACTGCCTCGC
Flk1in10 GATA-c mut_F	ctagGGGCTAGGTT*gggg*ACTGCCTCGC
Flk1in10 GATA-d_F	ctagTCCCACAGATAAGGAGGAGC

Flk1in10 GATA-d mut_F	ctagTCCCACAG*gg*AAGGAGGAGC
Flk1in10 ETS-a_F	ctagAAGGTCCTGAGGATACAGGAGGGA
Flk1in10 ETS-a mut_F	ctagAAGGTCCTGAG*cc*TACAGGAGGGA
Flk1in10 ETS-b_F	ctagATACAGGAGGGAAGCAGCTATTCT
Flk1in10 ETS-b mut_F	ctagATACAGGAGG*cc*AGCAGCTATTCT
Flk1in10 ETS-c_F	ctagTTCATAAATTGGATCGACAAGACA
Flk1in10 ETS-c mut_F	ctagTTCATAAATTG*cc*TCGACAAGACA
Flk1in10 ETS-d_F	ctagACTTAGTTCAAAGGAAGGTAAGGA
Flk1in10 ETS-d mut_F	ctagACTTAGTTCAAAG*cc*AGGTAAGGA
Flk1in10 ETS-e_F	ctagAGAAGGTAAGGAAACTTGGA
Flk1in10 ETS-e mut_F	ctagAGAAGGTAAG*cc*AACTTGGA
Flk1in10 ETS-f_F	ctagCTTTTGTTTAGGAAATGGCCAGCAG
Flk1in10 ETS-f mut_F	ctagCTTTTGTTTAG*cc*AATGGCCAGCAG
Flk1in10 ETS-g_F	ctagCAGCAGCAGAGGAAGAAACTCGGGT
Flk1in10 ETS-g mut_F	ctagCAGCAGCAGAG*cc*AGAAACTCGGGT
Flk1in10 ETS-h_F	ctagACAACAACAGGAAGTGGACTGC
Flk1in10 ETS-h mut_F	ctagACAACAACAG*cc*AGTGGACTGC
Flk1in10 ETS-i_F	ctagAACAGAAAGTGGAATGCTTGGG
Flk1in10 ETS-i mut_F	ctagAACAGAAAGTG*cc*ATGCTTGGG
Flk1in10 ETS-j_F	ctagGGCTGTTGTTTTCCCTAAGGACGT
Flk1in10 ETS-j mut_F	ctagGGCTGTTGTTT*gg*CCTAAGGACGT
Flk1in10 ETS-k_F	ctagTTTTGCTAAGGATGTCTGCACTT
Flk1in10 ETS-k mut_F	ctagTTTTGCTAAG*cc*TGTCTGCACTT
Flk1in10 ETS-l_F	ctagCCACCAGCACTTCCTCTGAGAGAT
Flk1in10 ETS-l mut_F	ctagCCACCAGCACT*gg*CTCTGAGAGAT
Flk1in10 RBPJ-a_F	ctagAGCTATTCTGGGAACAAGTCTCCA
Flk1in10 RBPJ-a mut_F	ctagAGCTATTC*c*GG*cc*AC*c*AGTCTCCA
Flk1in10 RBPJ-b_F	ctagACTTAGTTCAAAGGAAGGTAAG
Flk1in10 RBPJ-b mut_F	ctagACTTAGTT*tg*A*c*GGAAGGTAAG
Flk1in10 SOX-a_F	ctagAGTCTCCATTCATAAATTGGA
Flk1in10 SOX-a mut_F	ctagAGTCTCCAT*ggg*TAAATTGGA
Flk1in10 SOX-b_F	ctagAAGACAATTCAAGCTCACTTA
Flk1in10 SOX-b mut_F	ctagAAGACAATG*gg*AGCTCACTTA
Flk1in10 SOX-c_F	ctagTCACTTAGTTCAAAGGAAGGTA
Flk1in10 SOX-c mut_F	ctagTCACTTAGT*ggg*AAGGAAGGTA
Flk1in10 FOX-a,b_F	ctagATTTCAAAAACAACAACAGGAAGTGGAATG
Flk1in10 FOX-a,b mut a,b_F	ctagATTTCA*c*A*gg*C*g*ACA*g*C*c*GGAAGTGGAATG
Flk1in10 FOX-a,b mut a_F	ctagATTTCA*c*A*gg*C*g*ACAACAGGAAGTGGAATG
Flk1in10 FOX-a,b mut b_F	ctagATTTCAAAAAC*g*ACA*g*C*c*GGAACTGGAATG
ChIP Flk1in10 RBPJ-a F	caacagtgcgaaggtcctga
ChIP Flk1in10 RBPJ-a R	agcttgaattgtcttgtcgatcc
ChIP Flk1in10 RBPJ-b F	taggtttatcactgcctcgca
ChIP Flk1in10 RBPJ-b R	accacactgctcctccttatc

**Morpholinos**		
**Name**	MO sequence	Reference
GATA1ATG MO	CTGCAAGTGTAGTATTGAAGATGTC	4
GATA2a splice MO	CATCTACTCACCAGTCTGCGCTTTG	4
RBPJ MO	CAAACTTCCCTGTCACAACAGGCGC	6
scrambled MO	CCTCTTACCTCAGTTACAATTTATA	12
Sox 7 ATG MO	ACGCACTTATCAGAGCCGCCATGTG	5
Sox18 ATG MO	TATTCATTCCAGCAAGACCAACACG	5
Tnnt2 MO	CATGTTTGCTCTGATCTGACACGCA	7

Sequence of synthesized DNA fragments

Mouse Flk1in10 WT

TGCATGTCAAGATTTGACTTCTCTCTCGTTCAGGAGTGCCGGAAAGGGTCAGCCTCTGGTTA
TCACGTTCCTAGTGATAACCCTCGACACACTCGAACACTTCGCAGAACTTGGCGCCAATTAA
AAATAGATGCCTATACACAACAGTGCGAAGGTCCTGAGGATACAGGAGGGAAGCAGCTATTC
TGGGAACAAGTCTCCATTCATAAATTGGATCGACAAGACAATTCAAGCTCACTTAGTTCAAA
GGAAGGTAAGGAAACTTGGAAGCCATTGGGGCTTCTTAAAAGTCACCTCTCTGGGACGGACC
GACTGCGGGCTTTTGTTTAGGAAATGGCCAGCAGCAGAGGAAGAAACTCGGGTTTGCTATTT
CAAAAACAACAACAGGAAGTGGAATGCTTGGGGTGGTAGGTTGAAGGTGGTGGCTGTTGTTT
TCCCTAAGGATGTCTGCACTTGTGGTAGACCTTGATAAGCCTGGGCCTGAGACTCTCGAGGC
CTGGGCTAGGTTTATCACTGCCTCGCATCCGCCAGCACTTCCTCTGAGAGATGGACATTCCC
ACAGATAAGGAGGAGCAGTGTGGTCCTCTGCAGTCCACAGACAGAAGATGATCCGATGATTG
GCATCTAGCAAACGCAGCAAGTAGTATCCCTTTGGAGAGGAATCCACCAGGGCTAACTAAGG
AGAAAGAGCTGACTCGCATAGTGGGATACGGGGGAATGAGTCCAAGTACCACTGAAGGGGTA
ACCTTGGAGACAGCCTTCCGGGTTTCGACGGAACCCTGGCATGGCTTGGCAAGAAGGACGC
ACGATGGCAAGTGAAACAGA

Mouse Flk1in10 mut ETS-f,g,h,k,l

TGCATGTCAAGATTTGACTTCTCTCTCGTTCAGGAGTGCCGGAAAGGGTCAGCCTCTGGTTA
TCACGTTCCTAGTGATAACCCTCGACACACTCGAACACTTCGCAGAACTTGGCGCCAATTAA
AAATAGATGCCTATACACAACAGTGCGAAGGTCCTGAGGATACAGGAGGGAAGCAGCTATTC
TGGGAACAAGTCTCCATTCATAAATTGGATCGACAAGACAATTCAAGCTCACTTAGTTCAAA
GGAAGGTAAGGAAACTTGGAAGCCATTGGGGCTTCTTAAAAGTCACCTCTCTGGGACGGACC
GACTGCGGGCTTTTGTTTAG*cc*AATGGCCAGCAGCAGAG*cc*AGAAACTCGGGTTTGCTATTT
CAAAAACAACAACAG*cc*AGTGGAATGCTTGGGGTGGTAGGTTGAAGGTGGTGGCTGTTGTTT
TCCCTAAG*cc*TGTCTGCACTTGTGGTAGACCTTGATAAGCCTGGGCCTGAGACTCTCGAGGC
CTGGGCTAGGTTTATCACTGCCTCGCATCCGCCAGCACT*gg*CTCTGAGAGATGGACATTCCC
ACAGATAAGGAGGAGCAGTGTGGTCCTCTGCAGTCCACAGACAGAAGATGATCCGATGATTG
GCATCTAGCAAACGCAGCAAGTAGTATCCCTTTGGAGAGGAATCCACCAGGGCTAACTAAGG
AGAAAGAGCTGACTCGCATAGTGGGATACGGGGGAATGAGTCCAAGTACCACTGAAGGGGTA
ACCTTGGAGACAGCCTTCCGGGTTTCGACGGAACCCTGGCATGGCTTGGCAAGAAGGACGC
ACGATGGCAAGTGAAACAGA

Mouse Flk1in10 mut GATA-all
TGCATGTCAAGATTTGACTTCTCTCTCGTTCAGGAGTGCCGGAAAGGGTCAGCCTCTGGT*cc*
*c*CACGTTCCTAGT*ggg*GACCCTCGACACACTCGAACACTTCGCAGAACTTGGCGCCAATTAA
AAATAGATGCCTATACACAACAGTGCGAAGGTCCTGAGG*cc*ACAGGAGGGAAGCAGCTATTC
TGGGAACAAGTCTCCATTCATAAATTGGATCGACAAGACAATTCAAGCTCACTTAGTTCAAA
GGAAGGTAAGGAAACTTGGAAGCCATTGGGGCTTCTTAAAAGTCACCTCTCTGGGACGGACC
GACTGCGGGCTTTTGTTTAGGAAATGGCCAGCAGCAGAGGAAGAAACTCGGGTTTGCTATTT
CAAAAACAACAACAGGAAGTGGAATGCTTGGGGTGGTAGGTTGAAGGTGGTGGCTGTTGTTT
TCCCTAAGGATGTCTGCACTTGTGGTAGACCTTG*ggg*AGCCTGGGCCTGAGACTCTCGAGGC
CTGGGCTAGGTT*ccc*CACTGCCTCGCATCCGCCAGCACTTCCTCTGAGAGATGGACATTCCC
ACAG*ggg*AGGAGGAGCAGTGTGGTCCTCTGCAGTCCACAGACAGAAGATGATCCGATGATTG
GCATCTAGCAAACGCAGCAAGTAG*ccc*CCCTTTGGAGAGGAATCCACCAGGGCTAACTAAGG
AGAAAGAGCTGACTCGCATAGTGGG*ggg*CGGGGGAATGAGTCCAAGTACCACTGAAGGGGTA
ACCTTGGAGACAGCCTTCCGGGTTTCGACGGAACCCTGGCATGGCTTGGCAAGAAGGGACGC
ACGATGGCAAGTGAAACAGA

Mouse Flk1in10 mut GATA-b,c
TGCATGTCAAGATTTGACTTCTCTCTCGTTCAGGAGTGCCGGAAAGGGTCAGCCTCTGGTTA
TCACGTTCCTAGTGATAACCCTCGACACACTCGAACACTTCGCAGAACTTGGCGCCAATTAA
AAATAGATGCCTATACACAACAGTGCGAAGGTCCTGAGGATACAGGAGGGAAGCAGCTATTC
TGGGAACAAGTCTCCATTCATAAATTGGATCGACAAGACAATTCAAGCTCACTTAGTTCAAA
GGAAGGTAAGGAAACTTGGAAGCCATTGGGGCTTCTTAAAAGTCACCTCTCTGGGACGGACC
GACTGCGGGCTTTTGTTTAGGAAATGGCCAGCAGCAGAGGAAGAAACTCGGGTTTGCTATTT
CAAAAACAACAACAGGAAGTGGAATGCTTGGGGTGGTAGGTTGAAGGTGGTGGCTGTTGTTT
TCCCTAAGGATGTCTGCACTTGTGGTAGACCTTG*ggg*AGCCTGGGCCTGAGACTCTCGAGGC
CTGGGCTAGGTT*ccc*CACTGCCTCGCATCCGCCAGCACTTCCTCTGAGAGATGGACATTCCC
ACAGATAAGGAGGAGCAGTGTGGTCCTCTGCAGTCCACAGACAGAAGATGATCCGATGATTG
GCATCTAGCAAACGCAGCAAGTAGTATCCCTTTGGAGAGGAATCCACCAGGGCTAACTAAGG
AGAAAGAGCTGACTCGCATAGTGGGATACGGGGGAATGAGTCCAAGTACCACTGAAGGGGTA
ACCTTGGAGACAGCCTTCCGGGTTTCGACGGAACCCTGGCATGGCTTGGCAAGAAGGGACGC
ACGATGGCAAGTGAAACAGA

Mouse Flk1in10 mut RBPJ/mutSOX
TGCATGTCAAGATTTGACTTCTCTCTCGTTCAGGAGTGCCGGAAAGGGTCAGCCTCTGGTTA
TCACGTTCCTAGTGATAACCCTCGACACACTCGAACACTTCGCAGAACTTGGCGCCAATTAA
AAATAGATGCCTATACACAACAGTGCGAAGGTCCTGAGGATACAGGAGGGAAGCAGCTATTC
*c*GG*cc*AC*c*AGTCTCCAT*ggg*TAAATTGGATCGACAAG*g*C*g*AT*gg*GAGCTC*ggg*TAGT*ggg*AA
GGAAGGTAAGGAAACTTGGAAGCCATTGGGGCTTCTTAAAAGTCACCTCTCTGGGACGGACC
GACTGCGGGCTTTTGTTTAGGAAATGGCCAGCAGCAGAGGAAGAAACTCGGGTTTGCTATTT
CAAAAACAACAACAGGAAGTGGAATGCTTGGGGTGGTAGGTTGAAGGTGGTGGCTGTTGTTT
TCCCTAAGGATGTCTGCACTTGTGGTAGACCTTG*ata*AGCCTGGGCCTGAGACTCTCGAGGC
CTGGGCTAGGTT*tat*CACTGCCTCGCATCCGCCAGCACTTCCTCTGAGAGATGGCCAGGCTC
ACAGATAAGGAGGAGCAGTGTGGTCCTCTGCAGTCCACAGACAGAAGATGATCCGATGATTG
GCATCTAGCAAACGCAGCAAGTAGTATCCCTTTGGAGAGGAATCCACCAGGGCTAACTAAGG
AGAAAGAGCTGACTCGCATAGTGGGATACGGGGGAATGAGTCCAAGTACCACTGAAGGGGTA
ACCTTGGAGACAGCCTTCCGGGTTTCGACGGAACCCTGGCATGGCTTGGCAAGAAGGGACGC
ACGATGGCAAGTGAAACAGA

Mouse Flk1in10 mutRBPJ
TGCATGTCAAGATTTGACTTCTCTCTCGTTCAGGAGTGCCGGAAAGGGTCAGCCTCTGGTTA
TCACGTTCCTAGTGATAACCCTCGACACACTCGAACACTTCGCAGAACTTGGCGCCAATTAA
AAATAGATGCCTATACACAACAGTGCGAAGGTCCTGAGGATACAGGAGGGAAGCAGCTATTC

cGGccACcAGTCTCCATTCATAAATTGGATCGACAAGACAATTCAAGCTCACTTAGTTCAAA
GGAAGGTAAGGAAACTTGGAAGCCATTGGGGCTTCTTAAAAGTCACCTCTCTGGGACGGACC
GACTGCGGGCTTTTGTTTAGGAAATGGCCAGCAGCAGAGGAAGAAACTCGGGTTTGCTATTT
CAAAAACAACAACAGGAAGTGGAATGCTTGGGGTGGTAGGTTGAAGGTGGTGGCTGTTGTTT
TCCCTAAGGATGTCTGCACTTGTGGTAGACCTTGATAAGCCTGGGCCTGAGACTCTCGAGGC
CTGGGCTAGGTTTATCACTGCCTCGCATCCGCCAGCACTTCCTCTGAGAGATGGcCAggCtC
ACcGATAAGGAGGAGCAGTGTGGTCCTCTGCAGTCCACAGACAGAAGATGATCCGATGATTG
GCATCTAGCAAACGCAGCAAGTAGTATCCCTTTGGAGAGGAATCCACCAGGGCTAACTAAGG
AGAAAGAGCTGACTCGCATAGTGGGATACGGGGGAATGAGTCCAAGTACCACTGAAGGGGTA
ACCTTGGAGACAGCCTTCCGGGTTTCGACGGAACCCTGGCATGGCTTGGCAAGAAGGGACGC
ACGATGGCAAGTGAAACAGA

Chicken Flk1in10 WT
GCTGATCAACAAATCAGATGGATCACTGGCTACAGCCTACATTTCAGAAGGAAAAAGTATGT
TTCTTGAAATATTTCTATTAAATTGAACCCCATAACCTTCTGAGACCTTTGTGCTCTGTAAA
ATAAGAAGCCCCCAAGCTGAAAGGTGAATTCCCTAATTAATCTGCCTGTGTCAGCTCTATCA
GGGTGCTTGGAGGGAAGCAGCTATTCTGGGAACAAACCTCCATTCATAAATGCAAGATTCAA
GTTAATTTAGTTCAAAGGAAGACAGAGAGACATAGAAATCACTGAACTCCCTACAAGAATTT
CTTGAGGACAGTAGCCATTGTGTAGGAAGCAACCAGGCACCAAAGGAAGAAACTTGTGGAAA
TCACTTTAAAAACAACAACAGGAAAGAAGAATGCTGTTTTTTTTTCTTGACTTTTTTTTTTT
TTAAAGGAAATCTGGGCTTATGGCAGGCCCAGATAAGGGAAGCTGCGTGAATCTCAGTCAGG
CACTCTCTCTATCTTTCCCCAACATCAATCAGTGCACTTCCTTTGAGATACACTTTCACACA
GATAAAGAGCACTGTCACACTCAGTGCTTGAGCATAAGGAGAAAAAAACAACAACCACAAAG
GTCTGCAATTAGCATAAACAGCAAGTTTCCTGTGTTTGGGACTGAGCCCAATGGGAGAGCTG
ACAGGGGTGGGGGGCACAAAAATAGCTGTATTTGAAACCCATCATGGGTCAAGATGCCCTTG
AAGAAGGCTTGAAAGCAACAAGCCAGCGTTGCCAGCTCCTCACCACAGGTGGCCCTTGTCAA
CCCCCTCTCAACTCCCTGCCCATGAGTCAGCAGAGCCTGACTGCCTACTGAGTACAACGATC
TTCTTTGTTGGCAAGTGAAGTCATTTTGAGCCTACAGGTGTCATGAGCAAAATCTCTCATGA
CTTAAGAGGGAGCAAAACCAAATCCTAGGGCTGAATCCTTCAGTGTCCACCACTCTCTGTCC
AGCACATATGGCATAGGGAG

Mouse Flk1in10 mutGATA-a,b,d
TGCATGTCAAGATTTGACTTCTCTCTCGTTCAGGAGTGCCGGAAAGGGTCAGCCTCTGGTTA
TCACGTTCCTAGTGATAACCCTCGACACACTCGAACACTTCGCAGAACTTGGCGCCAATTAA
AAATAGATGCCTATACACAACAGTGCGAAGGTCCTGAGGccACAGGAGGGAAGCAGCTATTC
TGGGAACAAGTCTCCATTCATAAATTGGATCGACAAGACAATTCAAGCTCACTTAGTTCAAA
GGAAGGTAAGGAAACTTGGAAGCCATTGGGGCTTCTTAAAAGTCACCTCTCTGGGACGGACC
GACTGCGGGCTTTTGTTTAGGAAATGGCCAGCAGCAGAGGAAGAAACTCGGGTTTGCTATTT
CAAAAACAACAACAGGAAGTGGAATGCTTGGGGTGGTAGGTTGAAGGTGGTGGCTGTTGTTT
TCCCTAAGGATGTCTGCACTTGTGGTAGACCTTGgggAGCCTGGGCCTGAGACTCTCGAGGC
CTGGGCTAGGTTTATCACTGCCTCGCATCCGCCAGCACTTCCTCTGAGAGATGGACATTCCC
ACAGgggAGGAGGAGCAGTGTGGTCCTCTGCAGTCCACAGACAGAAGATGATCCGATGATTG
GCATCTAGCAAACGCAGCAAGTAGTATCCCTTTGGAGAGGAATCCACCAGGGCTAACTAAGG
AGAAAGAGCTGACTCGCATAGTGGGATACGGGGGAATGAGTCCAAGTACCACTGAAGGGGTA
ACCTTGGAGACAGCCTTCCGGGTTTCGACGGAACCCTGGCATGGCTTGGCAAGAAGGGACGC
ACGATGGCAAGTGAAACAGA

Mouse Flk1in10 mut GATA- a,b,d/RBPJ/SOX
TGCATGTCAAGATTTGACTTCTCTCTCGTTCAGGAGTGCCGGAAAGGGTCAGCCTCTGGTTA
TCACGTTCCTAGTGATAACCCTCGACACACTCGAACACTTCGCAGAACTTGGCGCCAATTAA
AAATAGATGCCTATACACAACAGTGCGAAGGTCCTGAGGccACAGGAGGGAAGCAGCTATTC
cGGccAcCAGTCTCCATgggTAAATTGGATCGACAAGgCgATggGAGCTCgggTAGTgggAA

GGAAGGTAAGGAAACTTGGAAGCCATTGGGGCTTCTTAAAAGTCACCTCTCTGGGACGGACC
GACTGCGGGCTTTTGTTTAGGAAATGGCCAGCAGCAGAGGAAGAAACTCGGGTTTGCTATTT
C*gg*AA*ggg*A*gg*ACAGGAAGTGGAATGCTTGGGGTGGTAGGTTGAAGGTGGTGGCTGTTGTTT
TCCCTAAGGATGTCTGCACTTGTGGTAGACCTTG*ggg*AGCCTGGGCCTGAGACTCTCGAGGC
CTGGGCTAGGTTTATCACTGCCTCGCATCCGCCAGCACTTCCTCTGAGAGATGG*cc*A*gg*C*t*C
A*c*CGgggAGGAGGAGCAGTGTGGTCCTCTGCAGTCCACAGACAGAAGATGATCCGATGATTG
GCATCTAGCAAACGCAGCAAGTAGTATCCCTTTGGAGAGGAATCCACCAGGGCTAACTAAGG
AGAAAGAGCTGACTCGCATAGTGGATACGGGGGAATGAGTCCAAGTACCACTGAAGGGGTA
ACCTTGGAGACAGCCTTCCGGGTTTCGACGGAACCCTGGCATGGCTTGGCAAGAAGGACGC
ACGATGGCAAGTGAAACAGA

Mouse Flk1in10 alt-mutGATA-b/RBPJ/SOX
TGCATGTCAAGATTTGACTTCTCTCTCGTTCAGGAGTGCCGGAAAGGGTCAGCCTCTGGTTA
TCACGTTCCTAGTGATAACCCTCGACACACTCGAACACTTCGCAGAACTTGGCGCCAATTAA
AAATAGATGCCTATACACAACAGTGCGAAGGTCCTGAGGATACAGGAGGGAAGCAGCTATT*a*
*aca*GAACAAGTCTCCAT*g*CATAAATTGGATCGACAAGACAATTC*g*AGCTCACTTAGTTC*gg*A
GGAAGGTAAGGAAACTTGGAAGCCATTGGGGCTTCTTAAAAGTCACCTCTCTGGGACGGACC
GACTGCGGGCTTTTGTTTAGGAAATGGCCAGCAGCAGAGGAAGAAACTCGGGTTTGCTATTT
CAAAAACAACAACAGGAAGTGGAATGCTTGGGGTGGTAGGTTGAAGGTGGTGGCTGTTGTTT
TCCCTAAGGATGTCTGCACTTGTGGTAGACCTTG*g*TAAGCCTGGGCCTGAGACTCTCGAGGC
CTGGGCTAGGTTTATCACTGCCTCGCATCCGCCAGCACTTCCTCTGAGAGATGGACATTC*tg*
*tg*AGATAAGGAGGAGCAGTGTGGTCCTCTGCAGTCCACAGACAGAAGATGATCCGATGATTG
GCATCTAGCAAACGCAGCAAGTAGTATCCCTTTGGAGAGGAATCCACCAGGGCTAACTAAGG
AGAAAGAGCTGACTCGCATAGTGGATACGGGGGAATGAGTCCAAGTACCACTGAAGGGGTA
ACCTTGGAGACAGCCTTCCGGGTTTCGACGGAACCCTGGCATGGCTTGGCAAGAAGGACGC
ACGATGGCAAGTGAAACAGA

Mouse Flk1in10 alt-mutGATA-b/RBPJ
TGCATGTCAAGATTTGACTTCTCTCTCGTTCAGGAGTGCCGGAAAGGGTCAGCCTCTGGTTA
TCACGTTCCTAGTGATAACCCTCGACACACTCGAACACTTCGCAGAACTTGGCGCCAATTAA
AAATAGATGCCTATACACAACAGTGCGAAGGTCCTGAGGATACAGGAGGGAAGCAGCTATaa
caGGAACAAGTCTCCATTCATAAATTGGATCGACAAGACAATTCAAGCTCACTTAGTTCAAA
GGAAGGTAAGGAAACTTGGAAGCCATTGGGGCTTCTTAAAAGTCACCTCTCTGGGACGGACC
GACTGCGGGCTTTTGTTTAGGAAATGGCCAGCAGCAGAGGAAGAAACTCGGGTTTGCTATTT
CAAAAACAACAACAGGAAGTGGAATGCTTGGGGTGGTAGGTTGAAGGTGGTGGCTGTTGTTT
TCCCTAAGGATGTCTGCACTTGTGGTAGACCTTGgTAAGCCTGGGCCTGAGACTCTCGAGGC
CTGGGCTAGGTTTATCACTGCCTCGCATCCGCCAGCACTTCCTCTGAGAGATGGACATTCCt
gtgGATAAGGAGGAGCAGTGTGGTCCTCTGCAGTCCACAGACAGAAGATGATCCGATGATTG
GCATCTAGCAAACGCAGCAAGTAGTATCCCTTTGGAGAGGAATCCACCAGGGCTAACTAAGG
AGAAAGAGCTGACTCGCATAGTGGATACGGGGGAATGAGTCCAAGTACCACTGAAGGGGTA
ACCTTGGAGACAGCCTTCCGGGTTTCGACGGAACCCTGGCATGGCTTGGCAAGAAGGACGC
ACGATGGCAAGTGAAACAGA

Mouse Flk1in10 alt-mutGATA-b/SOX
TGCATGTCAAGATTTGACTTCTCTCTCGTTCAGGAGTGCCGGAAAGGGTCAGCCTCTGGTTA
TCACGTTCCTAGTGATAACCCTCGACACACTCGAACACTTCGCAGAACTTGGCGCCAATTAA
AAATAGATGCCTATACACAACAGTGCGAAGGTCCTGAGGATACAGGAGGGAAGCAGCTATTC
TGGGAACAAGTCTCCATgCATAAATTGGATCGACAAGACAATTCgAGCTCACTTAGTTCggA
GGAAGGTAAGGAAACTTGGAAGCCATTGGGGCTTCTTAAAAGTCACCTCTCTGGGACGGACC
GACTGCGGGCTTTTGTTTAGGAAATGGCCAGCAGCAGAGGAAGAAACTCGGGTTTGCTATTT
CAAAAACAACAACAGGAAGTGGAATGCTTGGGGTGGTAGGTTGAAGGTGGTGGCTGTTGTTT
TCCCTAAGGATGTCTGCACTTGTGGTAGACCTTGgTAAGCCTGGGCCTGAGACTCTCGAGGC

CTGGGCTAGGTTTATCACTGCCTCGCATCCGCCAGCACTTCCTCTGAGAGATGGACATTCCC
ACAGATAAGGAGGAGCAGTGTGGTCCTCTGCAGTCCACAGACAGAAGATGATCCGATGATTG
GCATCTAGCAAACGCAGCAAGTAGTATCCCTTTGGAGAGGAATCCACCAGGGCTAACTAAGG
AGAAAGAGCTGACTCGCATAGTGGATACGGGGGAATGAGTCCAAGTACCACTGAAGGGGTA
ACCTTGGAGACAGCCTTCCGGGTTTCGACGGAACCCTGGCATGGCTTGGCAAGAAGGGACGC
ACGATGGCAAGTGAAACAGA

Kdr in situ probe
TCAGTCACGCGTCCGGCCAGTGCACGAGTCCAGCACTGAACGCGCAACAGGTCACTATTTCT
GTCGGCAAGGAGTTTTGCCCTTTATAGAGTCTGTTTCATTCGTTCTTACGGGGGTTGAGGA
TTTTAAACAGGCATCTAATAGAGCATAACTGTGGCCTGTATTCGTTATGAGGAGTTTTTGAT
TTTTGGTTTCATGTCTGGTCGGTCCAGCACTTCTGTCTGGCGATGGCAAAGACATCTTATGC
GCTTTTACTCCTCGACATCCTCCTCACCTTCAACGTGGCTAAAGCGATAGAGCTCAGGTTTG
TACCTGATCCTCCAACCTTGAACATCACTGAGAAGACTATCAAAATCAATGCTTCAGATACA
CTT

Supplemental References

1. Chi NC, Shaw RM, Val SD, Kang G, Jan LY, Black BL, Stainier DYR. Foxn4 directly regulates tbx2b expression and atrioventricular canal formation. *Genes Dev*. 2008;22:734–739.

2. De Val S, Anderson JP, Heidt AB, Khiem D, Xu S-M, Black BL. Mef2c is activated directly by Ets transcription factors through an evolutionarily conserved endothelial cell-specific enhancer. *Dev Biol*. 2004;275:424–434.

3. Sacilotto N, Monteiro R, Fritzsche M, Becker PW, Sanchez-del-Campo L, Liu K, Pinheiro P, Ratnayaka I, Davies B, Goding CR, Patient R, Bou-Gharios G, De Val S. Analysis of Dll4 regulation reveals a combinatorial role for Sox and Notch in arterial development. *Proc Natl Acad Sci USA*. 2013;110:11893–11898.

4. Galloway JL, Wingert RA, Thisse C, Thisse B, Zon LI. Loss of gata1 but not gata2 converts erythropoiesis to myelopoiesis in zebrafish embryos. *Dev Cell*. 2005;8:109–116. doi:10.1016/j.devcel.2004.12.001.

5. Cermenati S, Moleri S, Cimbro S, Corti P, Del Giacco L, Amodeo R, Dejana E, Koopman P, Cotelli F, Beltrame M. Sox18 and Sox7 play redundant roles in vascular development. *Blood*. 2008;111:2657–2666.

6. Sieger D, Tautz D, Gajewski M. The role of Suppressor of Hairless in Notch mediated signalling during zebrafish somitogenesis. *Mech Dev*. 2003;120:1083–1094.

7. Sehnert AJ, Huq A, Weinstein BM, Walker C, Fishman M, Stainier DYR. Cardiac troponin T is essential in sarcomere assembly and cardiac contractility. *Nat Genet*. 2002;31:106–110.

8. Jin S-W, Herzog W, Santoro MM, Mitchell TS, Frantsve J, Jungblut B, Beis D, Scott IC, D'Amico LA, Ober EA, Verkade H, Field HA, Chi NC, Wehman AM, Baier H, Stainier DYR. A transgene-assisted genetic screen identifies essential

regulators of vascular development in vertebrate embryos. *Dev Biol*. 2007;307:29–42.

9. Leslie JD, Ariza-McNaughton L, Bermange AL, McAdow R, Johnson SL, Lewis J. Endothelial signalling by the Notch ligand Delta-like 4 restricts angiogenesis. 2007;134:839–844.

10. Fouquet B, Weinstein BM, Serluca FC, Fishman MC. Vessel Patterning in the Embryo of the Zebrafish: Guidance by Notochord. *Dev Biol*. 1997;183:37–48.

11. Gering M, Patient R. Hedgehog signaling is required for adult blood stem cell formation in zebrafish embryos. *Dev Cell*. 2005;8:389–400.

12. Siekmann AF, Lawson ND. Notch signalling limits angiogenic cell behaviour in developing zebrafish arteries. *Nature*. 2007;445:781.
