# Supplementary figures and images for: An Intronic Flk1 Enhancer Directs Arterial-Specific Expression via RBPJ-Mediated Venous Repression
Source: Arterioscler Thromb Vasc Biol. 2016 May 25;36(6):1209–19. doi: 10.1161/ATVBAHA.116.307517 (PMC4894770; doi:10.1161/ATVBAHA.116.307517)

## Artery

Two enhancers drive Flk1 expression

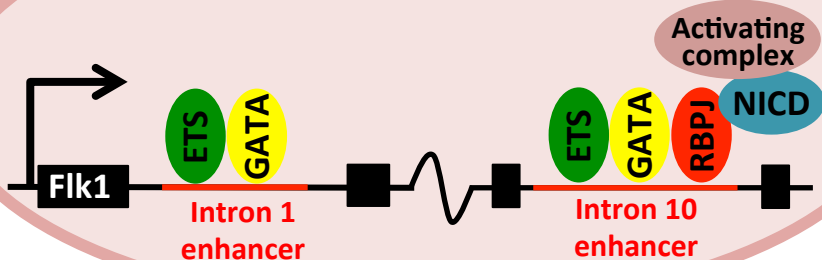

## Vein

Only one enhancer drives Flk1 expression

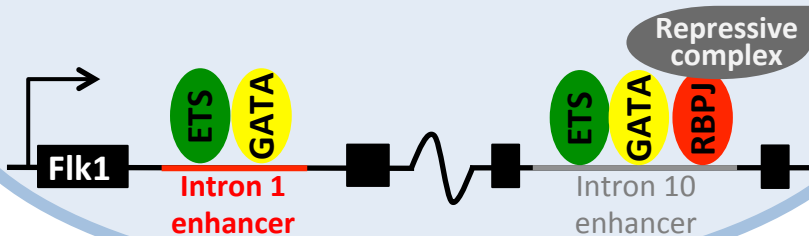

Supplement: Supplementary file 4 [file atv-36-1209-s004.pdf]
